# Phosphonate and Bisphosphonate Inhibitors of Farnesyl Pyrophosphate Synthases: A Structure-Guided Perspective

**DOI:** 10.3389/fchem.2020.612728

**Published:** 2021-01-06

**Authors:** Jaeok Park, Vishal R. Pandya, Sean J. Ezekiel, Albert M. Berghuis

**Affiliations:** ^1^Department of Biochemistry, Memorial University of Newfoundland, St. John's, NL, Canada; ^2^Department of Biochemistry, McGill University, Montreal, QC, Canada

**Keywords:** phosphonate, bisphosphonate, farnesyl pyrophosphate synthase, isoprenoid biosynthesis, structure-based drug design

## Abstract

Phosphonates and bisphosphonates have proven their pharmacological utility as inhibitors of enzymes that metabolize phosphate and pyrophosphate substrates. The blockbuster class of drugs nitrogen-containing bisphosphonates represent one of the best-known examples. Widely used to treat bone-resorption disorders, these drugs work by inhibiting the enzyme farnesyl pyrophosphate synthase. Playing a key role in the isoprenoid biosynthetic pathway, this enzyme is also a potential anticancer target. Here, we provide a comprehensive overview of the research efforts to identify new inhibitors of farnesyl pyrophosphate synthase for various therapeutic applications. While the majority of these efforts have been directed against the human enzyme, some have been targeted on its homologs from other organisms, such as protozoan parasites and insects. Our particular focus is on the structures of the target enzymes and how the structural information has guided the drug discovery efforts.

## Introduction

Phosphonates and bisphosphonates are chemically stable analogs of phosphates and pyrophosphates. Phosphate and pyrophosphate groups play numerous vital roles in the biochemistry of living organisms, and consequently, phosphonates and bisphosphonates constitute an important class of bioisosteres for medicinal chemists and chemical biologists (Elliott et al., 2012). The usefulness of this class of molecules has been highlighted by the COVID-19 pandemic. Phosphonate nucleotide analogs, such as tenofovir and cidofovir, can inhibit SARS-CoV-2 RNA polymerase (Jockusch et al., 2020) and may thus represent a much needed therapeutic weapon against this viral threat. Fosfomycin is another example of a clinically relevant phosphonate. Used primarily for urinary tract infections, fosfomycin is a bactericidal antibiotic that inhibits UDP-*N*-acetylglucosamine enolpyruvyl transferase, the enzyme responsible for the first committed step in peptidoglycan biosynthesis (Michalopoulos et al., 2011). Bisphosphonates, on the other hand, are particularly useful as inhibitors of enzymes involved in isoprenoid biosynthetic pathways. These compounds act by mimicking the pathway substrates that contain a pyrophosphate moiety. The pyrophosphate functionality promotes the solubility of these substrates and provides an efficient leaving group for condensation reactions.

**Figure 1:**
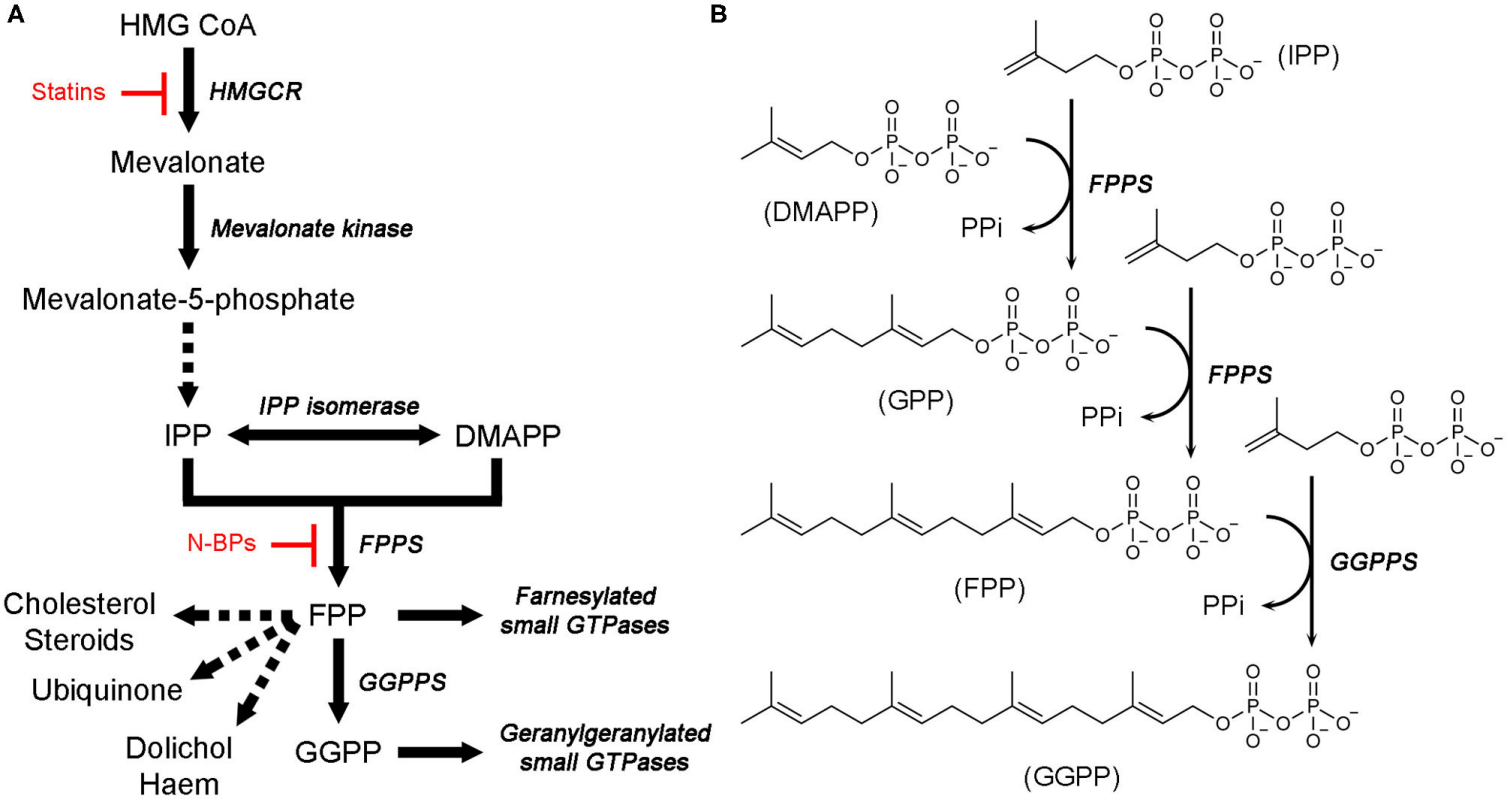
Mevalonate pathway and downstream metabolites. **(A)** An overview of the MVA pathway and downstream metabolites. Enzyme names are in italics. Dotted arrows represent multiple enzymatic steps. Sites of pharmacological intervention by current clinical drugs are indicated in red. **(B)** Catalytic steps of FPPS and GGPPS reactions.

Isoprenoids, also called terpenoids, refer to a large and diverse group of organic molecules containing isoprene moieties. Many essential metabolites belong to this group, for example, bile acids, vitamin D, steroid hormones, ubiquinone, haem A, dolichol, and isopentenyl adenine (Goldstein and Brown, 1990). Biosynthesis of all isoprenoids begins with the 5-carbon building blocks, isopentenyl pyrophosphate (IPP) and dimethylallyl pyrophosphate (DMAPP). In eukaryotes, these molecules are produced by a dedicated metabolic pathway called the mevalonate (MVA) pathway (Figure 1A). At the first branching point following this pathway, sits farnesyl pyrophosphate synthase (FPPS) (Figure 1A). This enzyme catalyzes the sequential elongation of DMAPP to geranyl pyrophosphate (GPP) and farnesyl pyrophosphate (FPP) (Figure 1B). FPP serves as a precursor to the downstream metabolites, such as those mentioned above. Alternatively, it can undergo another condensation reaction catalyzed by geranylgeranyl pyrophosphate synthase (GGPPS) to produce geranylgeranyl pyrophosphate (GGPP) (Figure 1B). FPP and GGPP function as farnesyl and geranylgeranyl lipid donors in protein prenylation (Figure 1A), a posttranslational modification essential for membrane localization of certain proteins. Prenylated proteins constitute up to 2% of the mammalian proteome and are best represented by the signaling proteins small GTPases (McTaggart, 2006).

Isoprenoid metabolites play key roles in various cellular activities, such as the formation of cell membranes (which requires cholesterol), production of sex hormones, electron transport (haem A and ubiquinone), glycoprotein biosynthesis (dolichol), modification of tRNAs (isopentenyl adenine), and cellular signaling (via membrane-targeted small GTPases), and thus their synthesis is a crucial prerequisite for the proper functioning of these processes. Therefore, enzymes responsible for the biosynthesis of isoprenoids have been of major pharmacological interest. The gateway enzyme of the MVA pathway, hydroxylmethylglutaryl coenzyme A reductase (HMGCR; Figure 1A), is the molecular target of the popular cholesterol-lowering agents statins (Endo, 2010). Inhibition of FPPS has been well established as the mechanism of action of another blockbuster class of drugs, nitrogen-containing bisphosphonates (N-BPs; Figure 1A), which are used to treat osteoclast-mediated bone resorption disorders (e.g., osteoporosis) (Russell, 2011). More recent interest has driven extensive drug discovery efforts focusing on the anticancer and antineurodegenerative effects of FPPS and GGPPS inhibition (Park et al., 2014; Waller et al., 2019). Enzymes of isoprenoid biosynthesis unique to pathogenic organisms are also being pursued as targets of new anti-infective agents (Masini and Hirsch, 2014).

In this review, we describe phosphonate- and bisphosphonate-based inhibitors of human FPPS that are current clinical agents or potential drug candidates. Our perspective is that of structure-based drug discovery. The structure of the enzyme and the binding modes of substrates and inhibitors are discussed, as well as key interactions that may be exploitable in inhibitor design. Structures of non-human FPPSs that are drug targets and the known inhibitors of these enzymes are also discussed.

## Human Farnesyl Pyrophosphate Synthase (hFPPS)

### Nitrogen-Containing Bisphosphonate (N-BP) Drugs

**Figure 2:**
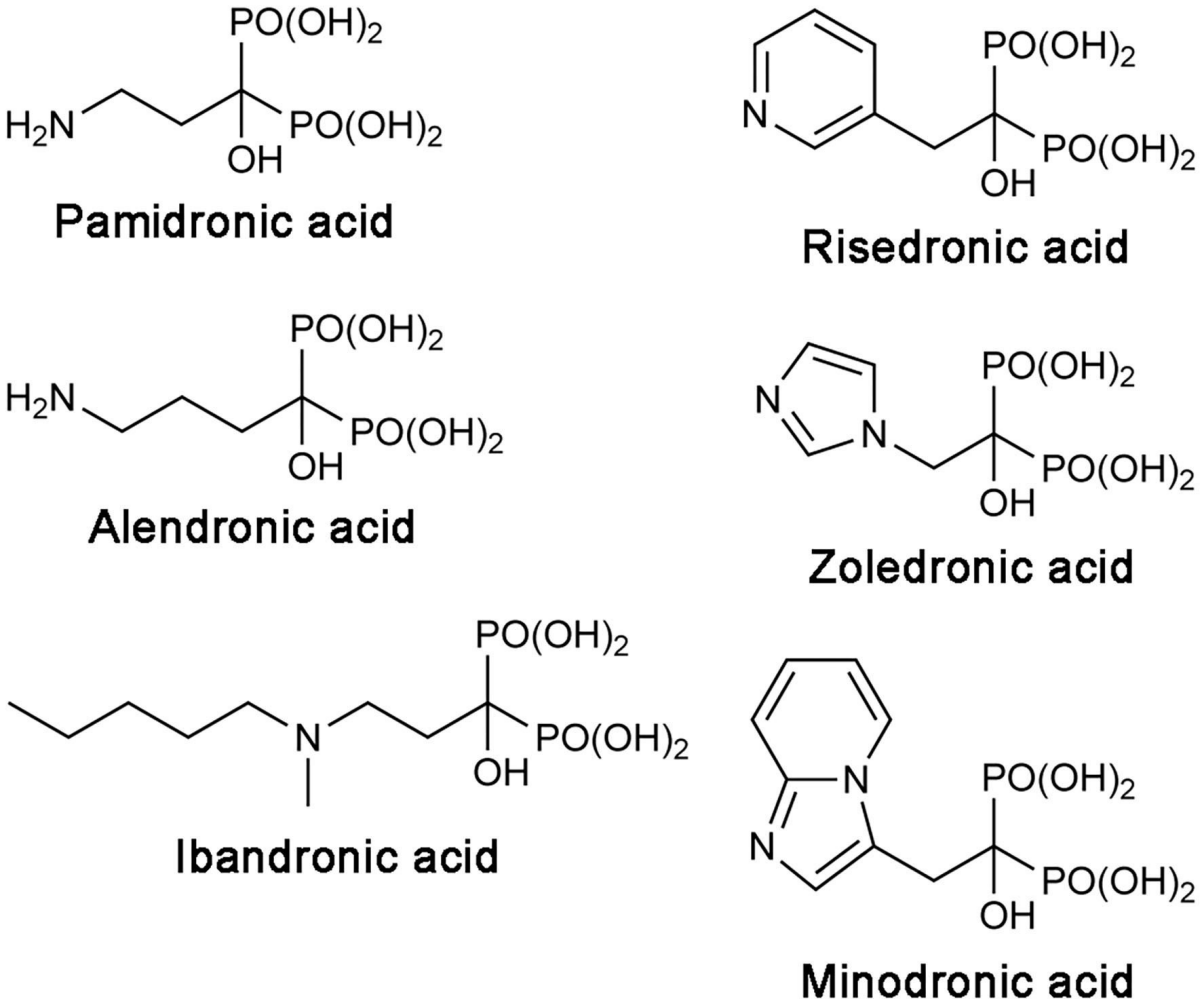
Structures of clinical N-BP drugs.

Bisphosphonate drugs are effective anti-bone resorption agents developed from the 1960s (Russell, 2011). They are used to treat bone lytic diseases, including osteoporosis, Paget's disease, and tumor-induced hypercalcemia. At the cellular level, all bisphosphonate drugs achieve their antiresorptive effects in the same way. They bind to bone, localizing preferentially at sites of resorption and mineral exposure, enter osteoclasts, the “bone-melting” cells, and induce apoptosis of these cells. At the molecular level, however, the mechanism of action differs between the first-generation bisphosphonate drugs and the more recent, N-BP drugs. The earlier drugs, such as clodronate and etidronate, are first metabolized intracellularly to cytotoxic ATP analogs, which then kills osteoclasts by inhibiting the mitochondrial enzyme adenine nucleotide translocase (Frith et al., 1997, 2001; Lehenkari et al., 2002). On the other hand, N-BPs directly inhibit their target enzyme, hFPPS (van Beek et al., 1999; Bergstrom et al., 2000), thereby blocking the synthesis of FPP and the downstream product GGPP (Figure 1A). This prevents the prenylation of small GTPases, such as members of the Ras superfamily, and consequently, the membrane targeting of these proteins, which is essential for osteoclast survival (Luckman et al., 1998; Benford et al., 1999; Coxon et al., 2000). N-BP drugs can be divided into two structural subgroups, the aliphatic bisphosphonates and the aromatic bisphosphonates. The former includes pamidronic acid, alendronic acid, and ibandronic acid, and the latter, risedronic acid, zoledronic acid, and minodronic acid (Figure 2).

### Structural Basis of hFPPS Function and Inhibition

**Figure 3:**
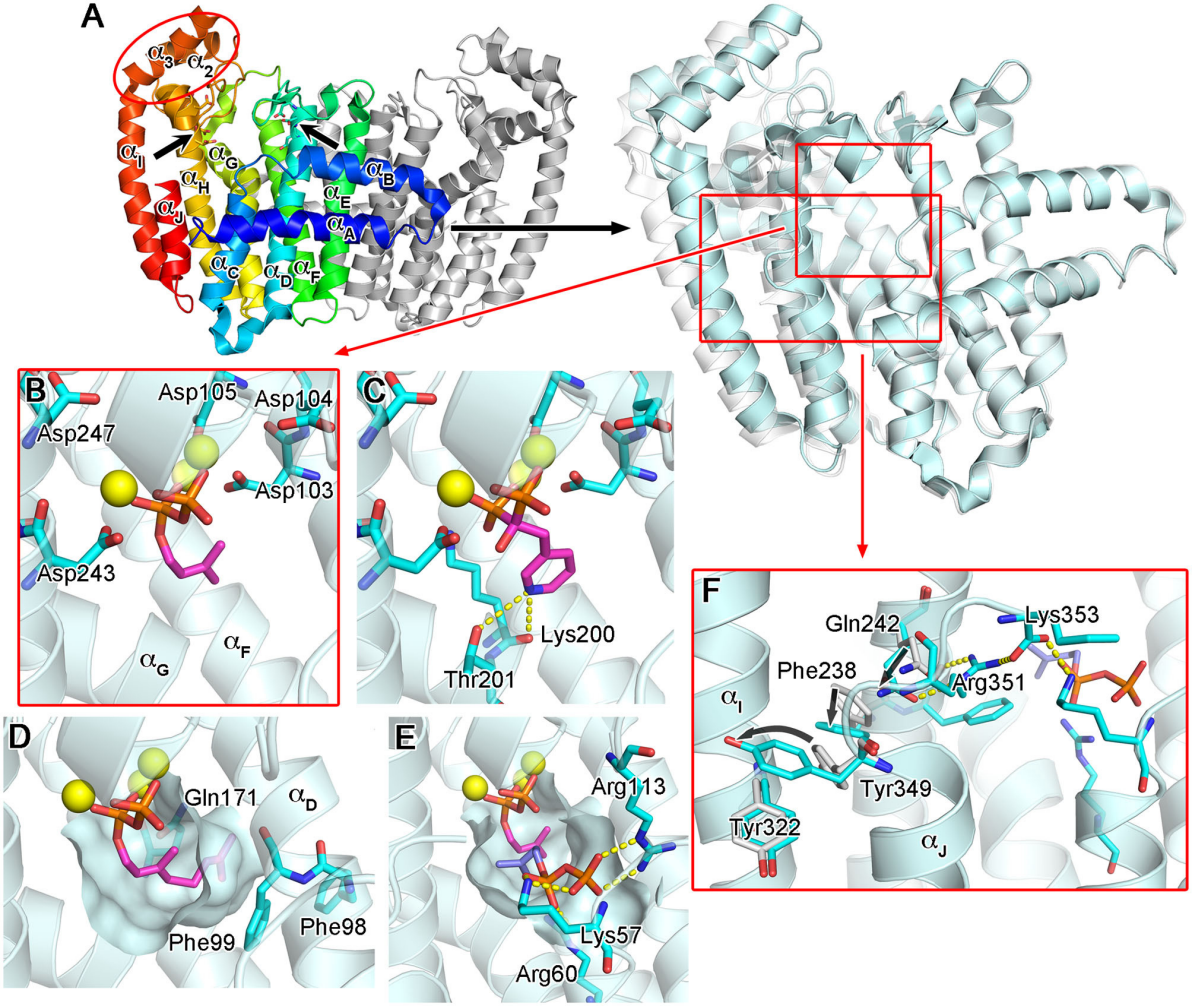
Overall structure and substrate/N-BP binding of human FPPS. **(A)** The overall structure of hFPPS. Left side: the homodimeric biological assembly in the unliganded open conformational state [Protein Data Bank (PDB) ID: 2F7M]. One subunit is represented in a rainbow color scheme (blue to red from the N- to C-terminus). The locations of the conserved aspartic acid-rich motifs are indicated by arrows. Right side: superimposed structures of hFPPS in the open (semi-transparent white; PDB ID: 2F7M) and fully closed (cyan; PDB ID: 4H5E) states. For clarity, only one subunit is displayed. **(B)** DMAPP (magenta) bound to the active site of hFPPS. The residues of the DDXXD motifs are in stick representation. Mg^2+^ ions are shown as yellow spheres. DMAPP was modeled into the PDB structure 4H5E based on an analog-bound *E. coli* FPPS structure (PDB ID: 1RQI). **(C)** The binding of risedronic acid (PDB ID: 1YV5). Yellow dashes indicate a bifurcated H-bond. **(D)** The hydrophobic pocket of the allylic substrate site in semi-transparent surface representation. Displayed in magenta is a modeled GPP molecule. **(E)** The IPP sub-pocket in the enzyme-substrates ternary complex (DMAPP in magenta and IPP in light purple). **(F)** The conformational cascade required for the C-terminal tail closure.

The molecular structure of hFPPS was first reported in 2006, independently by researchers from Novartis (Rondeau et al., 2006) and the Structural Genomics Consortium Oxford (Kavanagh et al., 2006). X-ray crystallographic studies revealed a homodimeric enzyme in an all α-helical fold (Figure 3A). Each monomeric subunit contains a central catalytic cavity shaped by 10 core helices (α_A_-α_J_; Figure 3A). Three additional helices (α_1_-α_3_) form a small peripheral domain (red ellipse, Figure 3A), which may function as a protein-protein interaction interface; notably, this insert is absent in the prokaryotic homologs (Hosfield et al., 2004; Schmidberger et al., 2015). The central cavity is lined with two aspartic acid-rich motifs (^103^DDIMD^107^ and ^243^DDYLD^247^), which face each other from opposite walls (Figure 3A). These motifs are essential for the binding of the allylic substrates (i.e., DMAPP and GPP) and are conserved across all homologs. The extended loop regions following the DDXXD motifs form the outer perimeter of the catalytic cavity. The dimerization of the subunits occurs through the formation of a helical bundle, with the helices α_E_ and α_F_ constituting the majority of the binding interface.

The binding of the allylic substrates requires the presence of metal ions. Coordinated between the aspartic acid residues of the DDXXD motifs and the pyrophosphate moiety of the bound substrate, three Mg^2+^ ions mediate the electrostatic interactions between these negatively charged groups (Figure 3B). Positively charged residues, Arg112, Lys200, and Lys257, which are also conserved, participate in this binding by forming direct salt bridges to the substrate pyrophosphate. The structural studies (Kavanagh et al., 2006; Rondeau et al., 2006) clearly revealed the binding mode of the N-BP drugs as well. These drugs act competitively with respect to the allylic substrates, binding to the same site in an analogous manner. In particular, the bisphosphonate moiety of the N-BP drugs mimics the pyrophosphate of the substrates, making identical Mg^2+^-mediated interactions to the DDXXD motifs (Figure 3C). The allylic substrate site also consists of a deep hydrophobic pocket, which accommodates the tail portion of the substrates (i.e., the dimethylallyl or geranyl group; Figure 3D) or the R_2_ side chain of the N-BP drugs. The pocket extends to the enzyme's dimerization interface, where it is closed off by the residues Ile143, Asn144, and Asn147 of the adjacent monomer. Other residues forming this pocket include Phe98, Phe99, and Gln171 (Figure 3D). In addition to providing a hydrophobic packing surface, the phenylalanine residues play a role in defining the final length of the isoprenyl product (Tarshis et al., 1996). Gln171 also contributes to the binding of the substrate tail group by providing stacking interactions through its planar side chain.

The binding of an allylic substrate or that of an N-BP drug leads to the formation of the second substrate (i.e., the homoallylic substrate IPP) binding site in hFPPS. The pyrophosphate of the allylic substrate, or the bisphosphonate group in the case of an N-BP, draws together the two DDXXD motifs via the Mg^2+^-mediated interactions, resulting in a rigid body motion that closes the enzyme. This conformational change significantly reduces the volume of catalytic cavity and fully shapes a sub-pocket for IPP to bind to Figure 3E. The surface of the IPP binding site is composed mainly of basic residues. Lys57, Arg60, and Arg113, which are also conserved, form direct salt bridges with the pyrophosphate of IPP when the substrate is bound (Figure 3E). Three other residues, Arg112, Lys257, and Arg351, participate in indirect binding interactions through water molecules. Additional interactions to the pyrophosphate group include a direct H-bond with Gln96 and water-mediated H-bonds with Asn59 and Glu93. Other conserved residues, Lue100 and Phe239, make hydrophobic contacts with the prenyl group of IPP. Together, these interactions position the homoallylic double bond of IPP within van der Waals distance to the first carbon (C-1) of the pre-bound allylic substrate.

The binding of IPP brings about yet another conformational change required for hFPPS catalysis: the four-residue C-terminal tail (^350^KRRK^353^), which exists as a flexible loop in the absence of bound IPP, folds over the substrate site and rigidifies. This conformational transition completely closes the enzyme's active site cavity, securing the substrates in position and shielding them from bulk water. The full closure of the enzyme protects the carbocation reaction intermediate (see below) from premature quenching, and thus, the C-terminal tail residues are essential for catalysis (Song and Poulter, 1994). The structural details of the tail closure have been well characterized (Park et al., 2012). Importantly, the guanidinium of Arg351 anchors to the core helix α_H_, while forming a salt bridge with the terminal carboxylic acid of Lys353 (Figure 3F). These interactions require a cascade of preceding conformational changes in the residues Gln242, Phe238, and Tyr349 (Figure 3F). Here, Tyr349 functions as a safety switch, which prevents any futile tail closure in the absence of bound IPP. With this residue locked in its “off” conformation, the downstream cascade is inhibited by steric hindrance.

**Figure 4:**
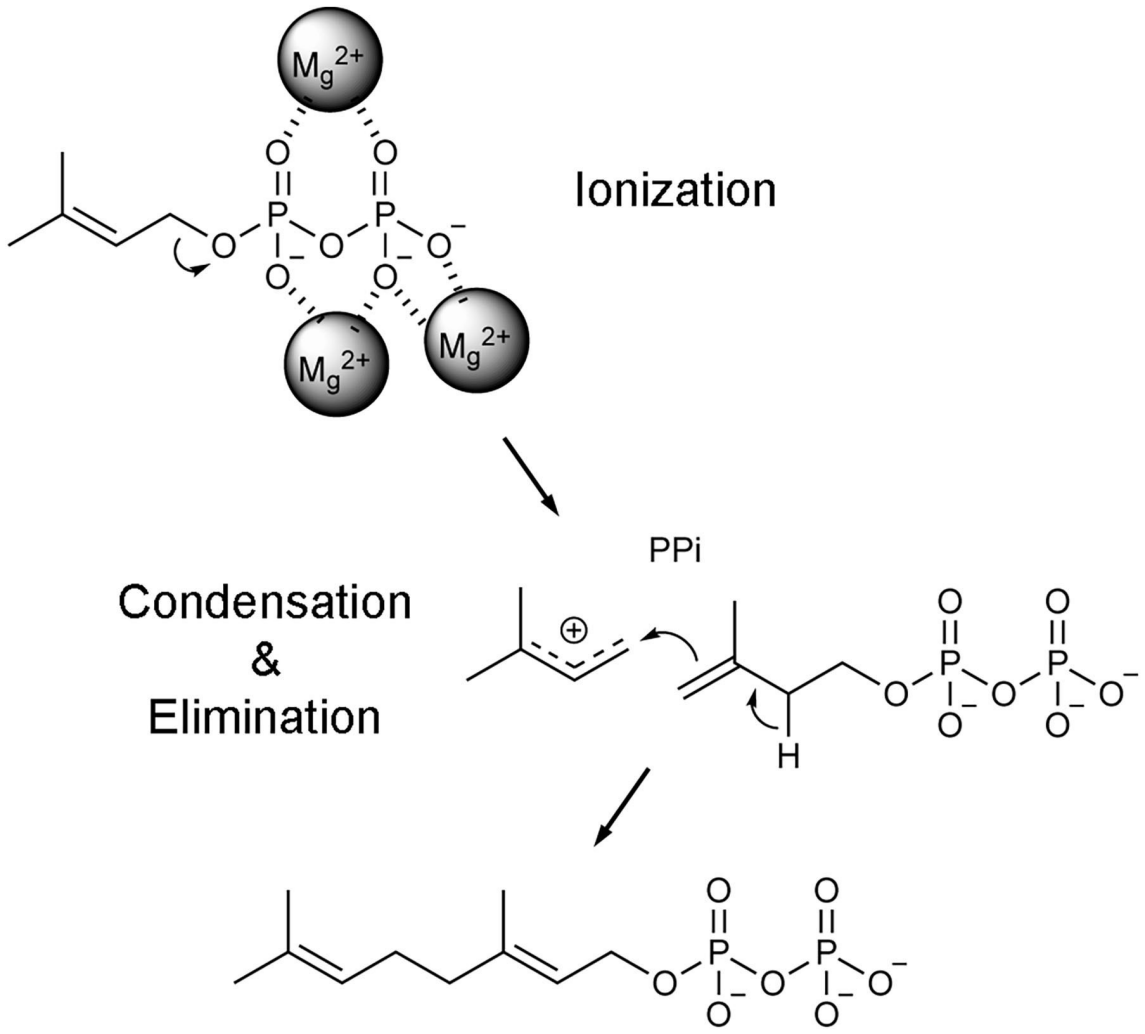
Catalytic mechanism of FPPS reaction. Only the first catalytic cycle (i.e., the condensation of DMAPP and IPP to produce GPP) is represented for simplicity. In the subsequent cycle, GPP is condensed with another unit of IPP to produce the final product FPP.

Once the hFPPS-substrates ternary complex is formed, catalysis proceeds in a three-step ionization-condensation-elimination reaction, in which the nucleophilic, tail double bond of IPP attacks the C-1 atom of the allylic substrate (i.e., DMAPP in the first cycle and GPP in the second) (Poulter et al., 1978). The initial step involves the ionization of the allylic substrate, which is triggered by the co-bound Mg^2+^ ions and produces a pyrophosphate anion and an allylic carbocation (Figure 4). The charge on the carbocation is distributed over its C-1, C-2, and C-3 atoms, stabilized by the main chain carbonyl of Lys200 and the side chain oxygen atoms of Thr201 and Gln240 (Hosfield et al., 2004). Once IPP captures the allylic carbocation intermediate, a concerted deprotonation step completes the chain elongation reaction (Figure 4).

Notably, heterocyclic N-BP drugs (viz., risedronic acid, zoledronic acid, and minodronic acid; Figure 2) can make similar interactions to those formed by the carbocation intermediate during catalysis (Martin et al., 1999). Under physiological conditions, their R_2_ side chain nitrogen (the namesake atom of these drugs) becomes protonated, forming a bifurcated H-bond with the main chain carbonyl of Lys200 and the side chain hydroxyl of Thr201 (Figure 3C). These interactions contribute significantly to the potency of N-BP drugs. For example, risedronic acid is 285-fold more potent than its phenyl analog in inhibiting hFPPS (IC_50_ = 5.7 vs. 1,626 nM) (Dunford et al., 2008).

Another factor that contributes to the high potency of N-BP drugs is the IPP-induced tail closure of hFPPS. As described above, N-BP binding at the allylic substrate site results in the conformation change that reshapes the enzyme's active site cavity and forms the IPP binding sub-pocket. Subsequent IPP binding leads to the formation of the enzyme-N-BP-IPP ternary complex, which unable to proceed through catalysis, stalls in the fully closed state (i.e., with the C-terminal tail closed). In this state, a competing substrate cannot easily replace the deeply buried inhibitor, and as a result, the binding of N-BPs is deemed nearly irreversible. This mechanism is at least partly responsible for the excellent efficacy of the current N-BP drugs.

### Human FPPS as an Anticancer Therapeutic Target

While the antiresorptive effects of N-BPs have long been known, the anticancer benefits of these drugs have started gaining attention more recently. A large number of pre-clinical studies have shown N-BPs' ability to suppress cancer proliferation, for example, for prostate (Iguchi et al., 2010; Mani et al., 2012), breast (Raikkonen et al., 2010; Dedes et al., 2012; Jiang et al., 2016), and colorectal (Notarnicola et al., 2004) cancers, human glioblastoma (Cimini et al., 2011), and multiple myeloma (Guenther et al., 2010). Clinical investigations have also provided evidence that N-BP drugs improve the survival of cancer patients. In a randomized trial involving >1,700 patients, supplementation of standard chemotherapy with zoledronic acid resulted in a statistically significant increase in the disease progression-free and overall survival of multiple myeloma patients (Morgan et al., 2010, 2012). For early-stage breast cancer, a large meta-analysis of 18,766 patient data concluded a positive correlation between bisphosphonate therapy and reduced risks of distant recurrence, bone recurrence, and mortality (Early Breast Cancer Trialists' Collaborative, 2015).

Most studies have attributed the observed anticancer effects of the N-BP drugs to the downregulation of protein prenylation, particularly that of small GTPases. Post-translational prenylation anchors small GTPases to cellular membranes, where they participate in a plethora of biological processes that are essential to cell survival, signaling, and proliferation (Takai et al., 2001). These processes play a critical role in oncogenesis and cancer metastasis as well, and numerous studies have shown a strong association between the inhibition of protein prenylation and cancer cell survival (Clendening et al., 2010; Sorrentino et al., 2014; Mullen et al., 2016) or metastasis (Dudakovic et al., 2011). This mechanism includes the downregulation of mutationally activated small GTPases, which would directly inhibit the proliferation of malignant cells harboring such proteins. Importantly, members of the Ras subfamily constitute the most frequently mutated oncogenic proteins in human cancers (Cox et al., 2014). For example, the combined results of cancer genome sequencing from two recent studies (Bolli et al., 2014; Lohr et al., 2014) have revealed a 42.6% frequency of either K-Ras or N-Ras coding mutations in multiple myeloma patients. Transforming mutations of Rho subfamily members (e.g., Rho, Rac, and Cdc42) are less frequent, but these proteins also play a key role in cancer formation and progression (Svensmark and Brakebusch, 2019). Intrinsically, small GTPases are either farnesylated (e.g., H/K/N-Ras) or geranylgeranylated (e.g., RhoA, Rac1, and Cdc42), but alternative prenylation (i.e., geranylgeranylation of proteins that are normally farnesylated and vice versa) is known to occur under certain conditions (Roberts et al., 2008; Berndt et al., 2011).

Another consequence of hFPPS inhibition is the intracellular accumulation of the enzyme's substrate, IPP. This accumulation leads to the production of an ATP analog, triphosphoric acid 1-adenosin-5'-yl ester 3-(3-methylbut-3-enyl) ester (ApppI; isopentenyl ester of ATP), which triggers apoptosis by inhibiting adenine nucleotide translocase (Monkkonen et al., 2006; Mitrofan et al., 2009). In addition, IPP can function as a phosphoantigen and activate Vγ2Vδ2 T cells (also known as Vγ9Vδ2 T cells), a subset of γδ T cells that can kill tumor cells (Morita et al., 2007). Therefore, inhibition of hFPPS provides an additional anticancer mechanism via the innate immune system. In support of this mechanism, knockdown of hFPPS by RNA interference has been shown to stimulate the tumor-killing activity of Vγ2Vδ2 T cells (Li et al., 2009; Wang et al., 2011). Intracellular accumulation of IPP and Vγ2Vδ2 T cell-mediated anticancer activity have also been observed in N-BP-treated animal models of breast cancer (Benzaid et al., 2012). Furthermore, evidence of immunostimulation (i.e., activation of γδ T cells and release of cytokines) induced by N-BP treatment has been observed in human patients with multiple myeloma (Kunzmann et al., 1999) and prostate cancer (Naoe et al., 2010). The activation of γδ T cells by N-BPs correlates specifically with the inhibition of FPPS and not of other isoprenoid synthesis enzymes, such as GGPPS (Zhang et al., 2010).

While there is enough evidence for the anticancer benefits of hFPPS inhibition, the current N-BP drugs' clinical utility is limited only to bone-related malignancies. Like their natural analog inorganic pyrophosphate (PPi), bisphosphonates have an extreme affinity for the hydroxyapatite bone mineral. N-BP drugs also feature a Cα-hydroxyl moiety (Figure 2). Commonly referred to as “bone hook,” this substituent maximizes the bone affinity of bisphosphonate compounds (Marma et al., 2007; Jahnke and Henry, 2010). As a result, systemically available N-BPs clear very rapidly from plasma, with a half-life of 1–2 h; in contrast, their half-life in bone ranges from 1 to 10 years, depending on the rate of bone turnover (Lin, 1996). In addition, due to the high charge density of the bisphosphonate moiety, which exists as a trianion under physiological conditions, N-BPs have poor membrane permeability. The oral absorption of these drugs is minimal (<3%), and they are not able to enter cells freely by simple diffusion (Lin, 1996). Macrophages and osteoclasts can internalize bisphosphonates by endocytosis, but the release of bisphosphonates from internalized vesicles requires endosomal acidification (Thompson et al., 2006). Consequently, the bioavailability of N-BPs is negligible in most non-skeletal tissues, thus compromising the full anticancer potential of these drugs.

### Exploratory Bisphosphonate Inhibitors of hFPPS

**Figure 5:**
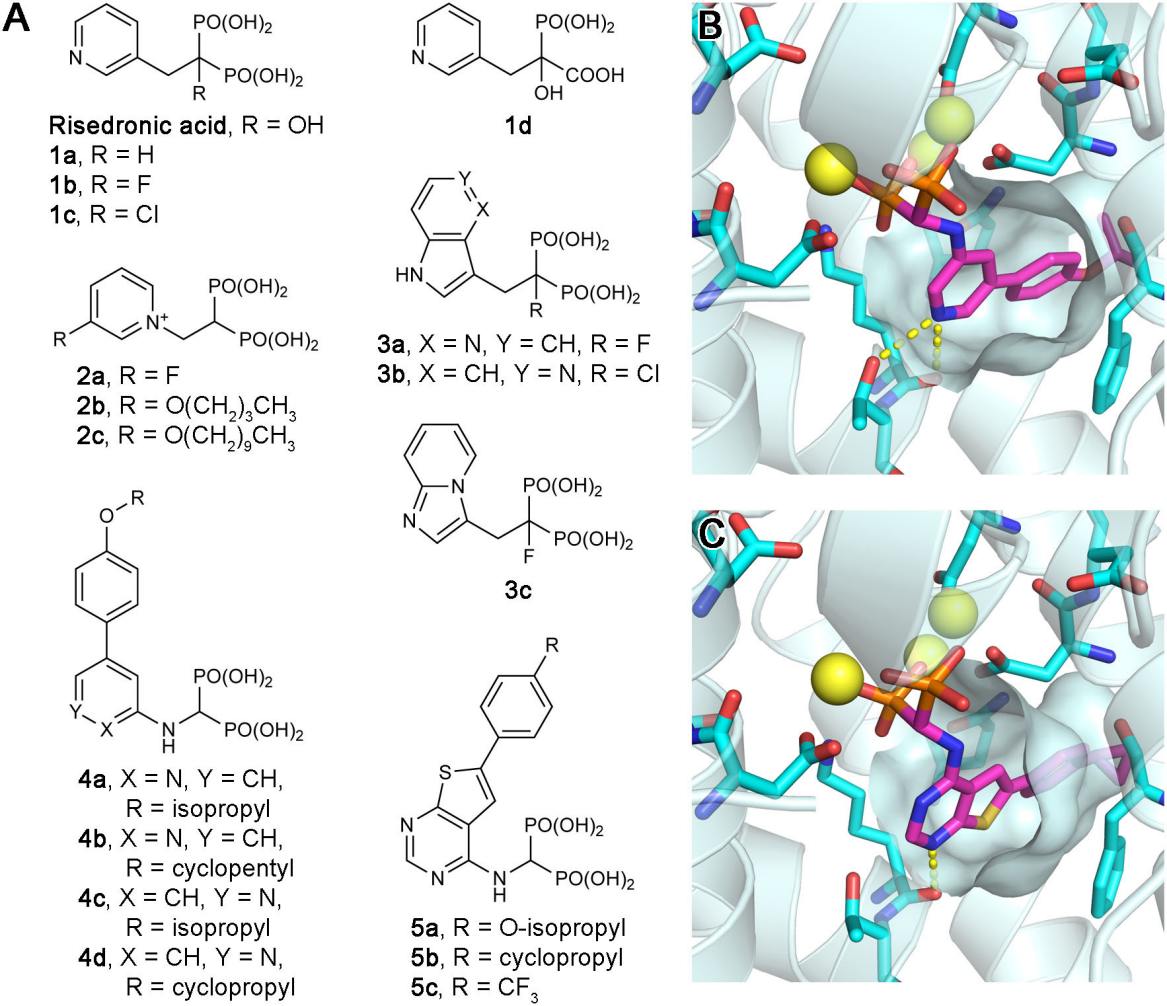
Exploratory bisphosphonate inhibitors of human FPPS. **(A)** Molecular structures of exploratory bisphosphonate inhibitors of hFPPS. **(B)** The binding of inhibitor **4c** (magenta; PDB ID: 4PVY). The residues of interest displayed as sticks are the same as in Figures 3B–D. **(C)** The binding of **5b** (magenta; PDB ID: 4L2X).

Owing to the potential anticancer benefits of hFPPS inhibition, there has been a great interest in identifying new inhibitors of hFPPS with favorable pharmacokinetic properties. Initial efforts were aimed at improving the current N-BP drugs by structural modification. Replacement of the Cα-hydroxyl group with a hydrogen (e.g., risedronic acid vs. **1a**; Figure 5A) or a halogen (e.g., **1b** and **1c**; Figure 5A) decreased the affinity of these compounds for bone, but also their potency for hFPPS inhibition (Marma et al., 2007). Replacement of one phosphonate group with a carboxylic acid (e.g., **1d**; Figure 5A) rendered these compounds essentially inactive (IC_50_ >200 μM) (Marma et al., 2007). Bisphosphonates with large lipophilic side chains have also been explored, including pyridinium deoxybisphosphonates (e.g., **2a**–**c**; Figure 5A) (Zhang et al., 2010), azaindoles and imidazopyridines (e.g., **3a**–**c**; Figure 5A) (Ebetino et al., 2010a,b, 2012), aminopyridines (e.g., **4a**–**d**; Figure 5A) (De Schutter et al., 2012; Lin et al., 2012; Park et al., 2017b), and thienopyrimidines (e.g., **5a**–**c**; Figure 5A) (Leung et al., 2013a,b). The key feature in the binding of these inhibitors is that their bulky lipophilic side chains can fully occupy the allylic substrate site hydrophobic cavity (Figures 5B,C). Some of these compounds showed improved pharmacokinetic properties (for targeting non-skeletal tissues). The intrinsic potency of the analogs **3b**, **3c**, and **4c** is equivalent to that of risedronic acid (i.e., similar IC_50_ values), but the bone affinity of these analogs is lower (Ebetino et al., 2010b; De Schutter et al., 2012). Evidence of improved membrane permeability has been observed, for example, with analog **4b**, whose cell-based potency is comparable to that of zoledronic acid (i.e., similar EC_50_ values) despite an eight-fold lower intrinsic potency (IC_50_ = 32 vs. 4.1 nM) (Lin et al., 2012). However, none of the bisphosphonate analogs explored to date has demonstrated systemic exposure sufficient for targeting non-skeletal tissues.

### Allosteric Inhibition of hFPPS

**Figure 6:**
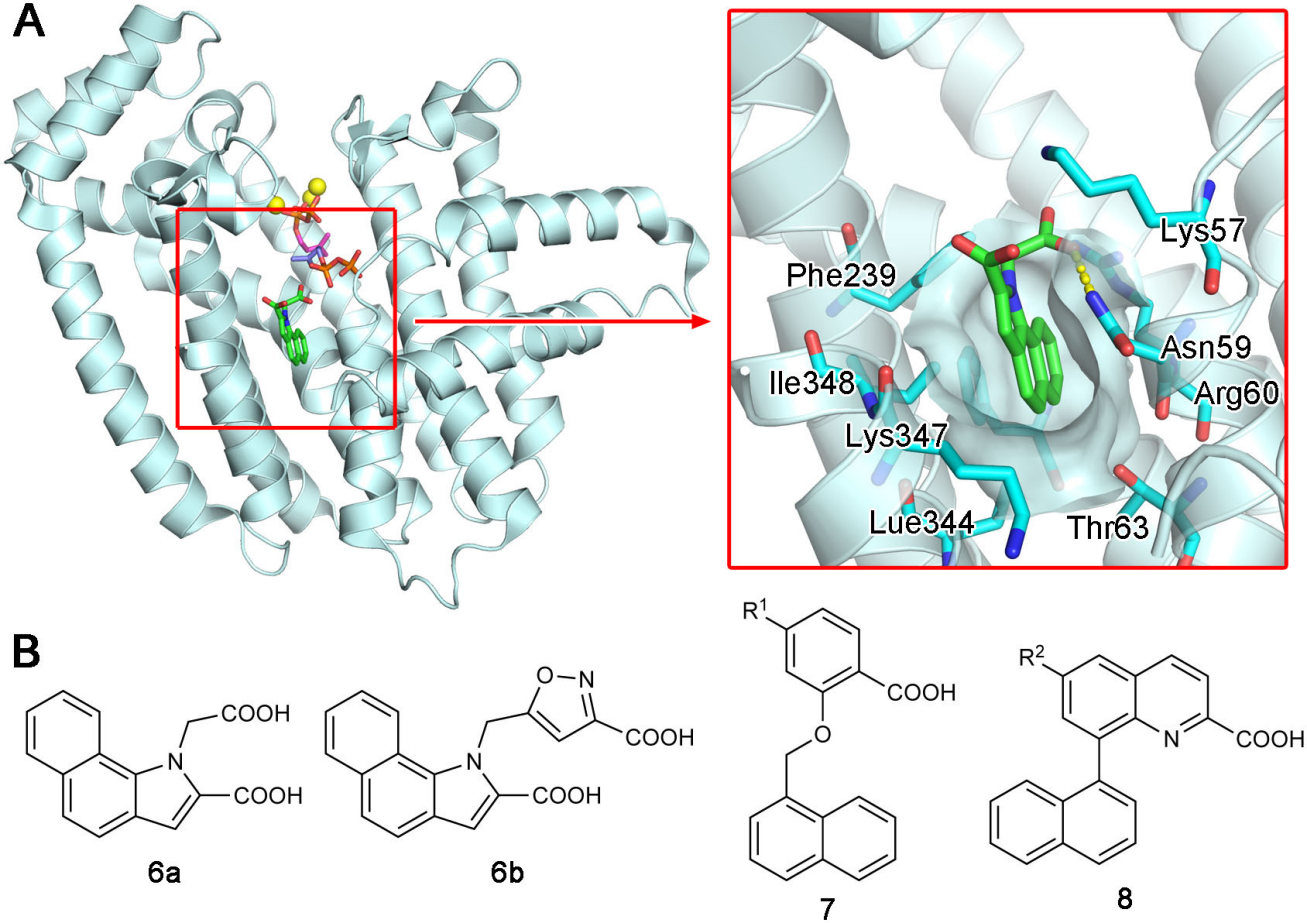
Discovery of non-bisphosphonate hFPPS inhibitors. **(A)** The binding of inhibitor **6a** (green; see panel B for its molecular structure) to the new druggable pocket of hFPPS (PDB ID: 3N6K). To provide a reference point, the binding sites for DMAPP (magenta), IPP (purple), and Mg^2+^ ions (yellow) are indicated in the overall structure via superposition. **(B)** Structures of non-bisphosphonate inhibitors of hFPPS. R^1^ and R^2^ in compounds 7 and 8 represent very broadly defined substituents, including hydrogen, halogen, and optionally substituted heterocyclic groups.

Increasing interest in the anticancer application of hFPPS inhibition, as well as the unfavorable bioavailability of bisphosphonates for this purpose, has fueled efforts to identify non-bisphosphonate inhibitors of hFPPS. In a fragment-based screening approach, a group from Novartis discovered such inhibitors that target a new druggable pocket in the enzyme (Jahnke et al., 2010). Found adjacent to the IPP binding site near the enzyme's C-terminal end (Figure 6A), this pocket is mostly hydrophobic. However, the opening of this pocket is highly polar on one side, lined with the side chain functional groups of Lys57, Asn59, and Arg60 (Figure 6A). Other residues forming this pocket include Thr63, Phe206, Phe239, Lue344, Lys347, and Ile348 (Figure 6A). The fragment hits were all based on a bicyclic heteroaromatic ring substituted with a carboxylic acid group. The final, optimized compounds, which showed sub-micromolar potency, consisted of a benzoindole scaffold and two carboxylic acids (**6a** and **6b**; Figure 6B). Co-crystal structures revealed the binding modes of these fragments and compounds. Their primary ring structure forms hydrophobic and aromatic interactions with the residues Tyr10, Phe206, Phe239, Lue344, and Lys347, whereas their carboxylic acid groups make electrostatic and polar interactions with Lys57, Asn59, and Arg60 (Figure 6A). In the meantime, the same Novartis group patented salicylic acid- and quinoline-based compounds (e.g., **7** and **8**; Figure 6B) as hFPPS inhibitors (Cotesta et al., 2011; Amstutz et al., 2012). Although the binding modes of these compounds were not reported at the time, based on their molecular structures, it was unlikely that they would function as active site inhibitors. It was revealed in a later publication that these series of inhibitors also bind to the new druggable pocket (Marzinzik et al., 2015).

While research for the therapeutic exploitation of the new druggable pocket continued, the intrinsic function of this pocket remained unknown for many years. An allosteric regulatory role was proposed when the pocket was first discovered, and potential biological effector molecules were tested based on the binding preference for lipophilic ligands containing a negatively charged functional group. However, neither cholesterol and bile acids, which are downstream metabolites of FPP, nor nucleotides and their analogs, such as ATP and GTP, were shown to inhibit hFPPS (Jahnke et al., 2010).

**Figure 7:**
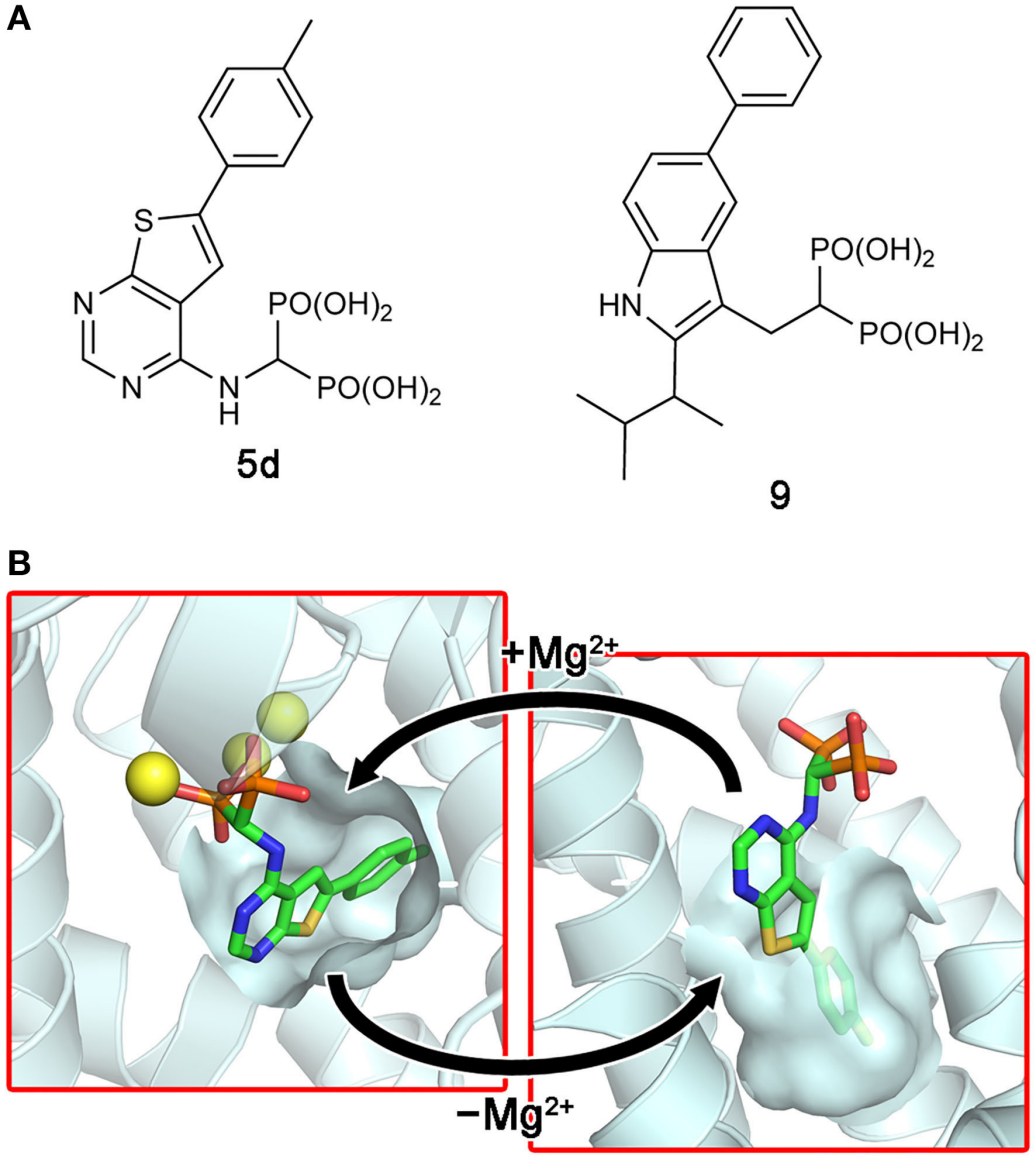
Bisphosphonate inhibitors with a dual binding mode. **(A)** Examples of thienopyrimidine and benzimidazole bisphosphonates. **(B)** The binding of inhibitor **5d** to the active site (left panel; PDB ID: 4JVJ) and the new druggable pocket (right; PDB ID: 4LPG) in the presence and absence of Mg^2+^ ions.

More recent screening efforts identified different series of compounds that also bind to the new druggable pocket of hFPPS (De Schutter et al., 2014; Gritzalis et al., 2015). Here it was discovered that bisphosphonates with a large lipophilic side chain, such as a thienopyrimidine or benzimidazole group (e.g., **5d** and **9**; Figure 7A), can have a dual binding mode: they bind to the allylic substrate site together with Mg^2+^ ions or to the newly found druggable pocket on their own (Figure 7B). This finding raised an interesting possibility. Similarly to how bisphosphonates bind to the allylic site by mimicking DMAPP or GPP, the natural ligand for the new pocket could also be a prenyl pyrophosphate.

**Figure 8:**
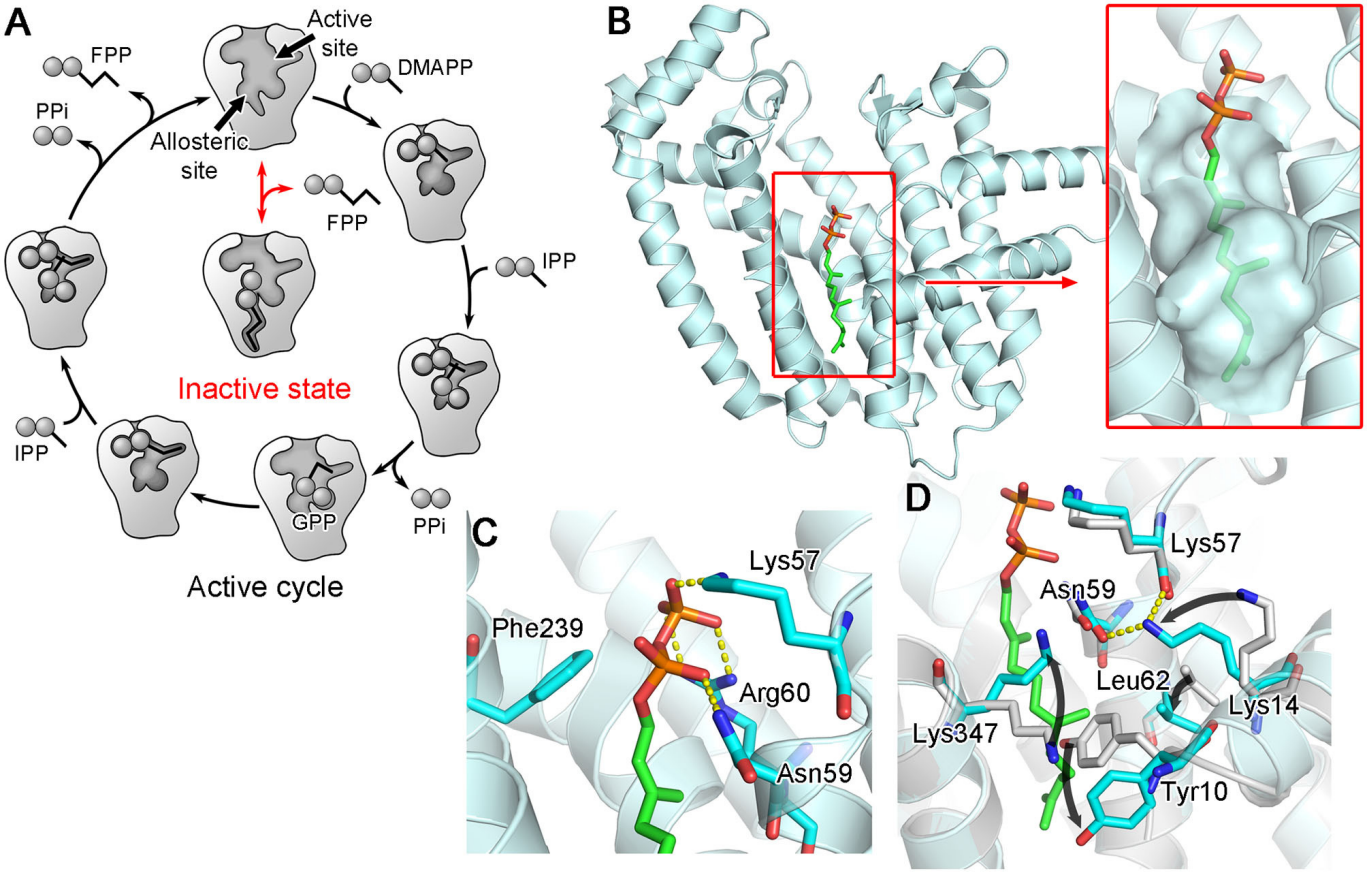
Allosteric binding of FPP to hFPPS. **(A)** A schematic representation of FPPS catalytic cycle and allosteric product inhibition. FPP binding locks the enzyme in its open, inactive state. **(B)** A crystal structure showing FPP bound to the allosteric pocket (PDB ID: 5JA0). **(C)** Residues making direct interactions with the pyrophosphate moiety. **(D)** Conformational changes induced by FPP binding. Superimposed in gray is the structure of hFPPS in the unliganded state (PDB ID: 2F7M).

A follow-up study indeed showed that FPP, none other than the product of hFPPS itself, can bind to the new pocket and inhibit the enzyme (Park et al., 2017c). The crystallographic analysis clearly elucidated the mechanism of inhibition: bound at the new site, FPP functions as a molecular wedge that prevents the closing of the enzyme, a conformational transition necessary for substrate binding and catalysis (Figure 8). This allostery, therefore, represents a negative product feedback mechanism. In retrospect, FPP binding at the allosteric pocket is not surprising, as this pocket shows an optimal architecture to bind a long hydrocarbon ligand with a negatively charged head group. The inner surface of this pocket provides hydrophobic and aromatic interactions to the prenyl tail of FPP (Figure 8B inset). The basic residues at the pocket opening clasp the pyrophosphate head via salt bridges and polar interactions; in addition, Phe239 forms a quadrupole-charge interaction with the pyrophosphate (Figure 8C). However, this binding involves an induced-fit mechanism that expands the pocket and reshapes its surface for better steric complementarity with the ligand (Figure 8D). This conformational change could not be predicted from the previously available structures.

### Monophosphonate Inhibitors of hFPPS

**Figure 9:**
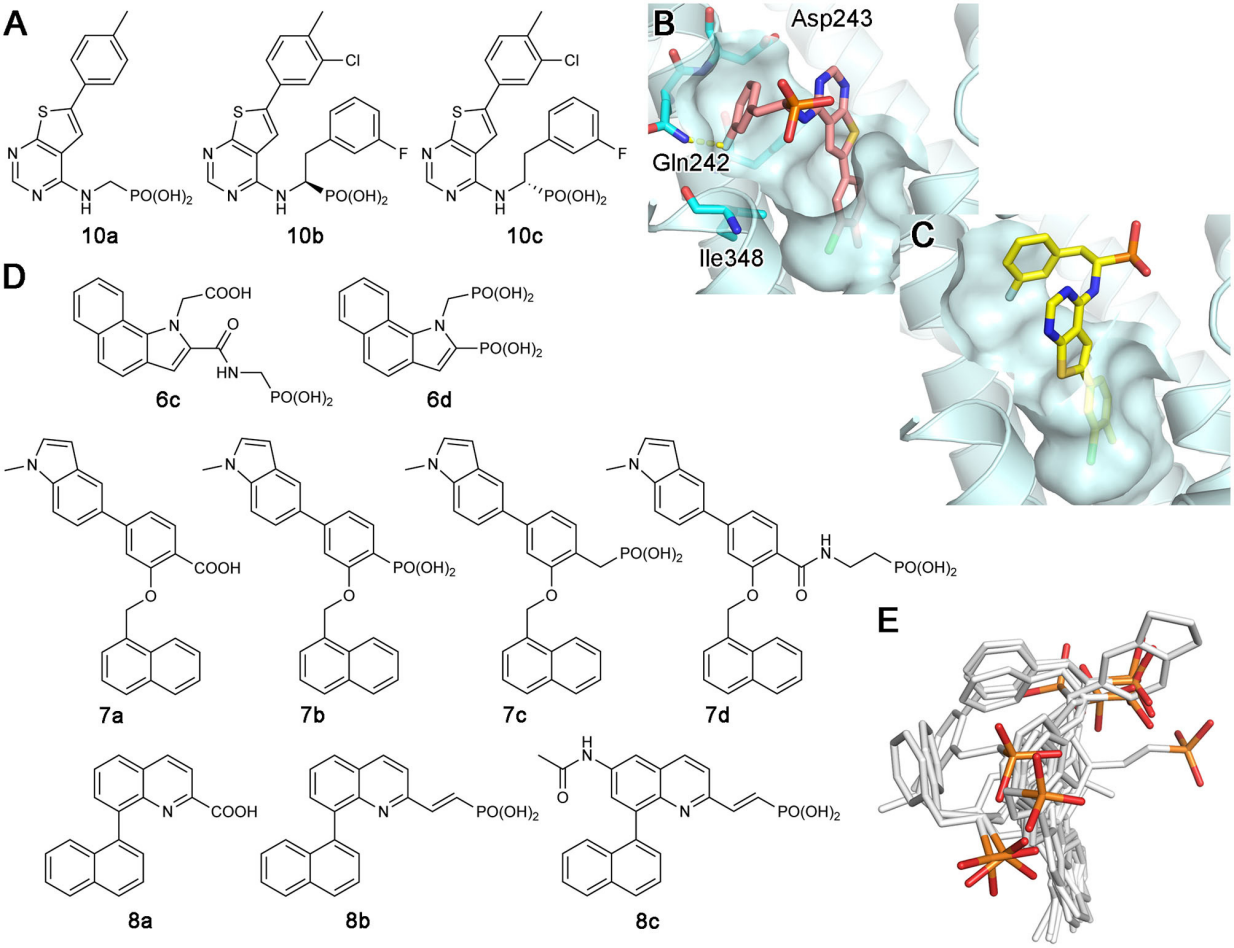
Examples and binding modes of allosteric inhibitors of hFPPS. **(A)** Thienopyrimidine-based monophosphonate inhibitors of hFPPS. **(B)** The binding of inhibitor **10b** at the allosteric pocket (PDB ID: 6N83). **(C)** The binding of **10c** (PDB ID: 6N82). **(D)** Structures of benzoindole-, salicylic acid-, and quinoline-based inhibitors of hFPPS. **(E)** Superimposed thienopyrimidine-, salicylic acid-, and quinoline-based monophosphonate inhibitors in their binding poses. Protein structures are omitted. The phosphorus and oxygen atoms of the phosphonate groups are colored in orange and red, respectively. All other atoms are colored in white.

Continued drug discovery efforts have identified many structurally diverse inhibitors that act at the allosteric site of hFPPS. Of particular interest here are the monophosphonate compounds. In structural remodeling of the promiscuously binding thienopyrimidine bisphosphonates (e.g., **5d**; Figure 7A), truncation of one phosphonate moiety led to a new series of inhibitors that bind exclusively to the allosteric site of the enzyme (e.g., **10a**; Figure 9A) (De Schutter et al., 2014; Park et al., 2017a). Further optimization of the monophosphonate compounds identified an analog with nanomolar *in vitro* potency (**10b**; Figure 9A) (Feng et al., 2019). Crystallographic studies have revealed how the newly introduced functional groups provide additional contributions to the binding of this compound. The 3-chloro substituent on the C-6 tolyl ring allows the tolyl group to fit more tightly into the allosteric pocket cavity (Figure 9B). The 3-fluoro substituent on the Cα benzyl ring plays a similar role. The halo-substitution results in the burial of the benzyl group in a small groove formed by the residues Phe239, Gln242, Asp243, and Ile348, where the benzyl group participates in a π-stacking interaction with the aromatic ring of Phe239, and the fluorine atom in a non-classical H-bond with the side chain amide of Gln242 (Figure 9B). Notably, the α-aminophosphonate moiety that characterizes this series of inhibitors has a chiral center (i.e., the Cα atom), and this chirality also affects the binding mode of these compounds significantly. For example, compound **10c** (Figure 9A), the (*S*)-enantiomer of **10b**, binds to the enzyme in a different orientation (Figure 9C) and has a two-fold lower inhibitory potency (IC_50_ = 0.54 vs. 1.1 μM).

The Novartis group also explored monophosphonate as a functionality on their allosteric inhibitors (Jahnke et al., 2015). Their strategy was to replace an existing carboxylic acid with either a phosphonate group or a phosphonate with a spacer, such as a methylene bridge (Figure 9D). The effect of such substitution is not straightforward. For the benzoindole and salicylic acid inhibitors, the monophosphonate analogs **6c** and **7b** (Figure 9D) are less potent (IC_50_ = 0.4 and 0.52 μM, respectively) than their respective parent compounds **6a** (Figure 6B) and **7a** (Figure 9D) (IC_50_ = 0.2 and 0.021 μM, respectively). In the quinoline series, however, the monophosphonate **8b** (Figure 9D; IC_50_ = 0.04 μM) is more potent than its parent compound **8a** (Figure 9D; IC_50_ = 1 μM). The NMR-based *in vitro* bone binding assay also produced interesting results (Jahnke et al., 2015). The salicylic acid parent compound **7a** and its simplest phosphonate analog **7b** did not show any bone binding activity. However, clear bone binding was observed for analog **7c** (Figure 9D), which contains a methylene spacer, and bone affinity was even stronger when the spacer was longer, as in analog **7d** (Figure 9D). A similar trend was observed with the benzoindole inhibitors **6a** (Figure 6B), **6c**, and **6d** (Figure 9D). The findings of this study suggest that while the presence of a phosphonate group alone does not indicate bone binding for allosteric FPPS inhibitors, bone affinity can be introduced to these compounds by adding a flexible linker together with the phosphonate group. Furthermore, the bone affinity of these compounds may even be tunable by changing the length of the linker. FPPS inhibitors with variable degrees of bone affinity could provide unique and useful pharmacological opportunities.

An intriguing observation with the monophosphonate allosteric inhibitors is that when the crystal structures showing these compounds are superimposed, the phosphonate groups of these compounds do not overlay very well (Figure 9E). The polycyclic cores of these compounds generally occupy the same space in the allosteric pocket, all engaged in π-stacking interactions with Asn59, Phe206, and/or Phe239. On the contrary, the phosphonate groups of these compounds do not have a consensus binding mode, each making different interactions to other specific residues. Further, phosphonate groups of some of these compounds are fully exposed to bulk solvent and do not seem to make any contact with the enzyme (e.g., **10b** and **6c**). On the basis of this observation, it was postulated that the phosphonate moiety in some allosteric inhibitors could be removed without significantly affecting their binding affinity for hFPPS (Park et al., 2017a). However, efforts to completely remove the phosphonate moiety has been unsuccessful thus far, while replacing it with another negatively charged group such as a carboxylic acid could be tolerated (Feng et al., 2019). In view of the fact that the allosteric pocket of hFPPS has evolved to bind FPP, a pyrophosphate-containing molecule, the requirement for a negatively charged functionality in an allosteric inhibitor fully makes sense. A potentially important role of this negatively charged group may be in interacting with the enzyme's KRRK C-terminal tail. Unstructured in the absence of bound IPP and composed of charged and flexible “disorder-promoting” residues, the C-terminal tail of hFPPS represents an intrinsically disordered protein region (Uversky, 2019). One of the key features of intrinsically disordered protein regions is that their structural plasticity allows them to interact with multiple partners in different binding modes. Located adjacent to the allosteric pocket, and although not “visible” in any of the crystal structures showing an allosterically bound inhibitor, the KRRK tail may play a significant role in attracting negatively charged small molecules to this pocket.

### Prodrugs of hFPPS Inhibitors

One way to overcome the poor membrane permeability of phosphate- or phosphonate-containing drugs is to use a prodrug strategy (Hecker and Erion, 2008; Wiemer and Wiemer, 2015). In this approach, the acidic oxygen atoms of the phosphate/phosphonate pharmacophore are capped with metabolically labile protecting groups to produce a neutrally charged prodrug molecule. With increased lipophilicity, the prodrug efficiently diffuses across the cell membrane into the cytosol. Removal of the capping groups by corresponding metabolic enzymes activates the prodrug into its acidic forms, which can then act on its intracellular target.

**Figure 10:**
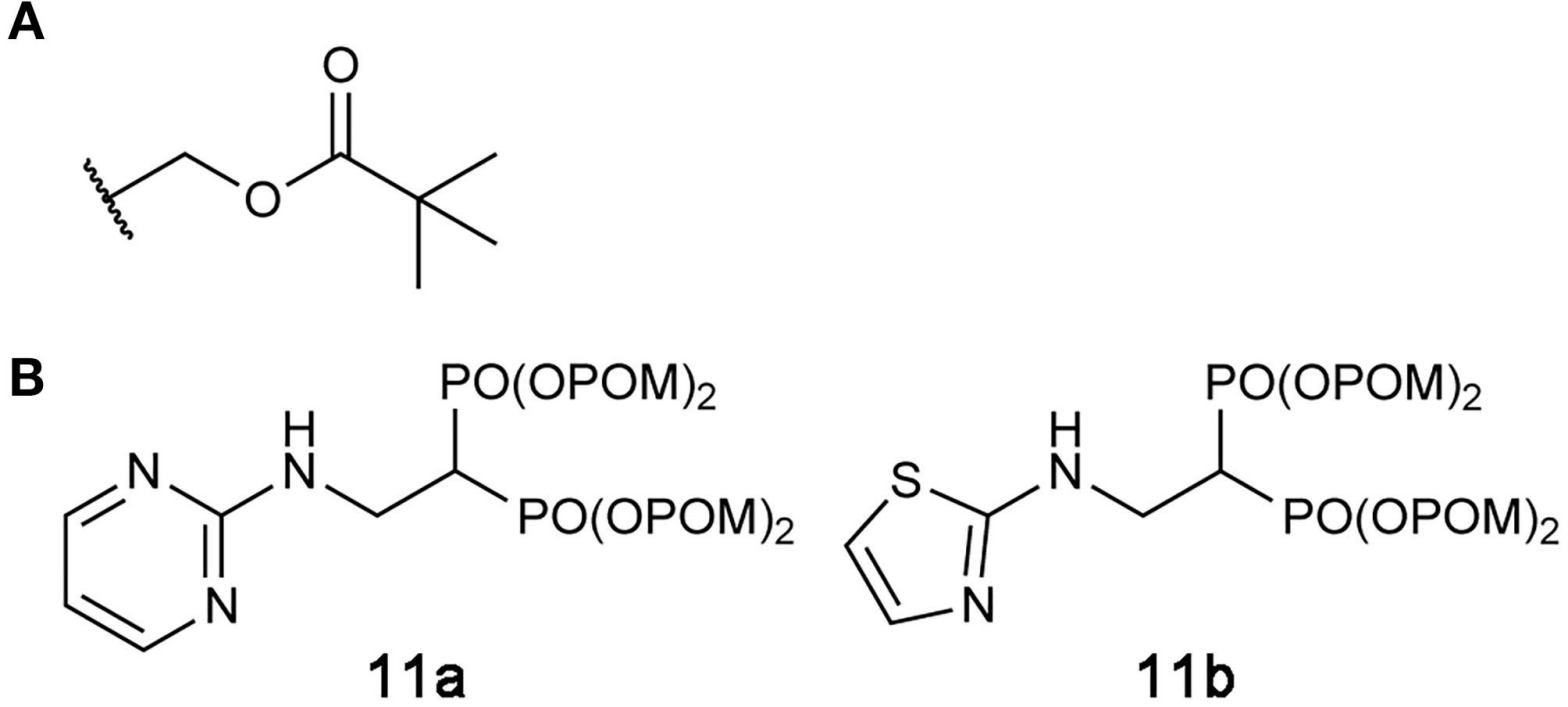
Prodrugs to target hFPPS. **(A)** The structure of the pivaloyloxymethyl (POM) group. **(B)** Examples of POM-protected N-BP prodrugs.

Exciting progress has been made in the development of hFPPS-targeting prodrugs in recent years. By masking phosphonate moieties with pivaloyloxymethyl (POM) groups (Figure 10A), a strategy also proven useful for other enzyme inhibitors (Majer et al., 2016; Matthiesen et al., 2018), Matsumoto and colleagues identified N-BP prodrugs that can inhibit hFPPS with significantly improved cellular potency (Matsumoto et al., 2016). The most active prodrug, compound **11b** (Figure 10B), inhibited the *in vitro* growth of various types of tumor cells at nanomolar concentrations—with the mean EC_50_ values of 240 and 770 nM for hematopoietic cancer cells and non-hematopoietic solid tumor cells, respectively. The EC_50_ of zoledronic acid, in comparison, was on average 796-fold higher for hematopoietic cells and 27-fold higher for non-hematopoietic cells. Inhibition of small GTPase prenylation, as well as the intracellular accumulation of IPP, has been confirmed as a downstream effect of the prodrug treatment (Tanaka et al., 2017; Okuno et al., 2020). Furthermore, the treatment of just 10–30 nM compound **11b** was enough to sensitize lung cancer cells to Vγ2Vδ2 T cells, thereby inducing significant lysis of the cancer cells by the immune cells (Okuno et al., 2020). These results suggest POM-protected prodrugs of N-BPs as a promising new form of cancer therapy; however, they are now too hydrophobic for use in clinical settings. Selective and efficient delivery systems are needed for these highly cytotoxic compounds to minimize their accumulation in healthy tissues and the resulting side effects. Other potential side effects may result from the production of formaldehyde, which is toxic and carcinogenic, as a metabolic byproduct of POM cleavage (Wiemer and Wiemer, 2015).

## Trypanosomatid FPPS

### FPPS as an Antiparasitic Target

Parasitic diseases account for one of the world's most significant human health problems. It is estimated that more than 3 billion people are afflicted by parasitic diseases, such as the protozoan infections trypanosomiasis and leishmaniasis (Feasey et al., 2010). American trypanosomiasis, also known as Chagas disease, is a potentially terminal illness caused by the parasite *Trypanosoma cruzi* (Lidani et al., 2019). Another dangerous parasite in the Trypanosoma family is *Trypanosoma brucei*, which results in African trypanosomiasis, commonly known as African sleeping sickness (Ponte-Sucre, 2016). Leishmaniasis, on the other hand, is caused by not one, but more than 20 different species of parasites from the genus *Leishmania*. (Georgiadou et al., 2015). The most common form of this disease is cutaneous leishmaniasis, which is caused by species like *Leishmania major* and *Leishmania mexicana*. Responsible species for the most lethal form, visceral leishmaniasis, include *Leishmania donovani*. Belonging to the neglected tropical disease group that the World Health Organization has prioritized to combat against, Chagas disease, African sleeping sickness, and leishmaniasis all occur under conditions that are intimately linked to poverty. There are only a few drugs available for the treatment of these diseases, and they suffer from limitations such as inefficacy, side effects, high costs, or impracticality for field use (Kaiser et al., 2015). Together with the emergence of resistance against current treatments (Capela et al., 2019), these shortcomings make the search for new and more effective drugs a high priority.

Studies have confirmed the presence of the MVA pathway in a range of parasitic protozoan species (Coppens and Courtoy, 1996). The pathway is essential for the biosynthesis of ergosterols unique to these organisms and is thus of vital importance to their survival. Consequently, parasitic FPPS has been proposed as a potential target for the treatment of protozoan afflictions (Martin et al., 2001). Being potent inhibitors of human FPPS, N-BPs have received particular attention. Acidocalcisomes of protozoan parasites, which are calcium-storing organelles that are also rich in pyrophosphates and polyphosphates, are thought to be able to accumulate N-BPs, thereby facilitating their antiparasitic action (Docampo and Moreno, 2008). In addition, as they are clinical drugs for the long-term treatment of bone disorders, their safety profile is well known. They have the advantage that they are relatively easy and inexpensive to synthesize as well. A number of *in vitro* assays have demonstrated that certain N-BPs and their analogs effectively inhibit the growth of protozoan parasites, including *T. cruzi, T. brucei*, and *L. donovani* (Martin et al., 2001, 2002; Szajnman et al., 2008; Rosso et al., 2011). Furthermore, animal studies have shown that risedronate can significantly increase the survival of mice infected with *T. cruzi* (Bouzahzah et al., 2005) and *L. donovani* (Yardley et al., 2002). Similarly, another N-BP, pamidronate, was found to be effective against *L. mexicana in vivo* (Rodriguez et al., 2002).

### Structures of Trypanosomatid Protozoan FPPSs

**Figure 11:**
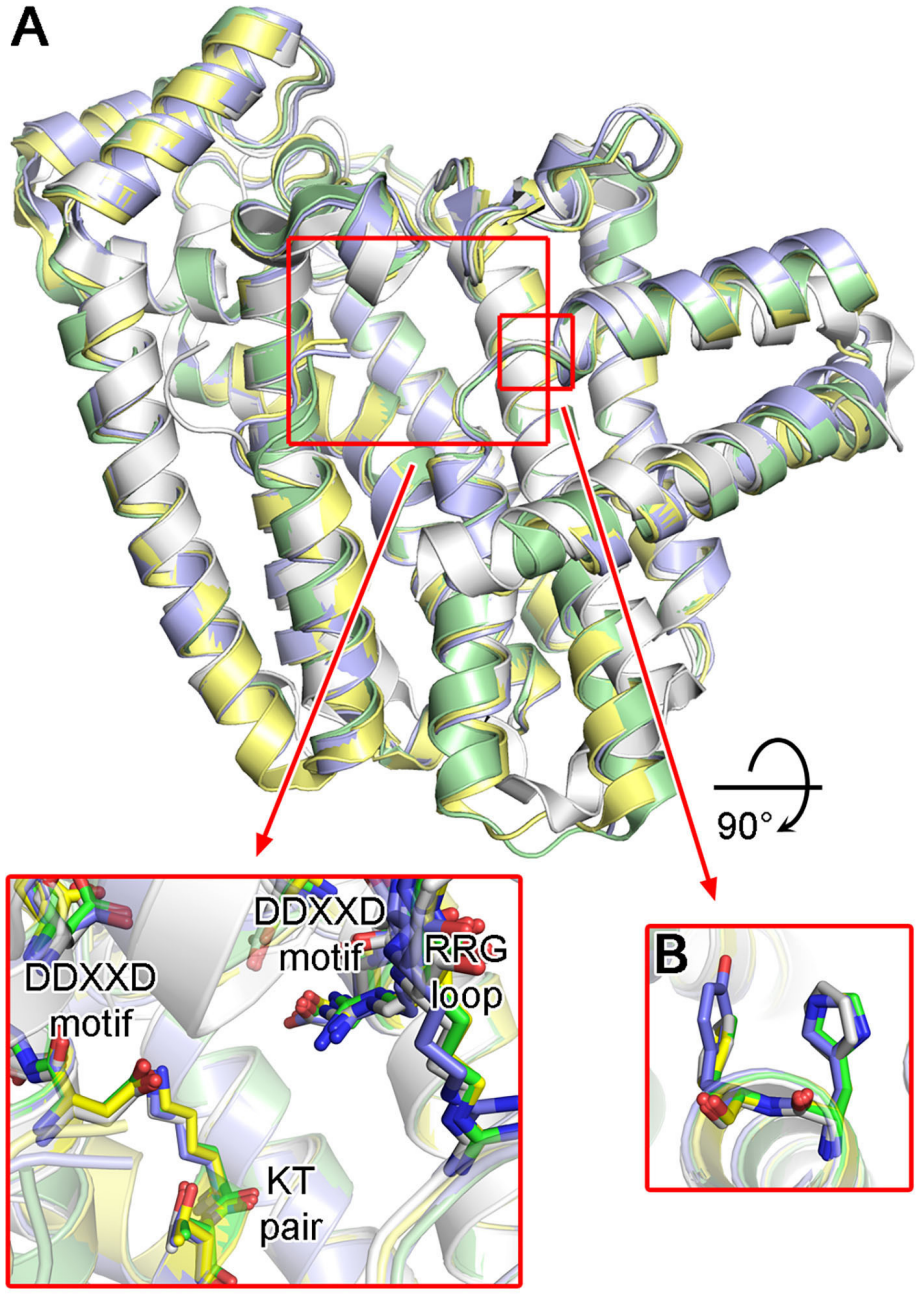
Structures of trypanosomatid FPPS. **(A)** Superimposed structures of *T. cruzi* (green; PDB ID: 4DWG), *T. brucei* (light purple; PDB ID: 4RXD), *L. major* (yellow; PDB ID: 4JZX) and human FPPS (white; PDB ID: 4H5E). Conserved residues of the active site are displayed in the inset. **(B)** Aromatic residues of the allylic substrate site hydrophobic pocket.

Structures of protozoan FPPSs have been extensively studied, specifically those of the disease-causing species *T. cruzi* (Gabelli et al., 2006; Huang et al., 2010; Aripirala et al., 2012), *T. brucei* (Mao et al., 2006; Cao et al., 2008; Zhang et al., 2009; Yang et al., 2015), and *L. major* (Aripirala et al., 2014). Despite only ~40% sequence identity, the overall structure of the protozoan FPPSs is fundamentally identical to that of the human homolog (Figure 11A). The active site residues are particularly well conserved, including the DDXXD motifs, the KT pair, and the RRG loop region (Figure 11A). A notable difference is in the residues of the allylic site hydrophobic pocket that are involved in the product length determination. Often referred to as the “capping” phenyls, Phe98 and Phe99 play this role in the human enzyme (see Section “Structural Basis of hFPPS Function and Inhibition”). However, in all three protozoan homologs, Phe98 is replaced by a histidine residue (Figure 11B). Phe99, on the other hand, is retained in *L. major* FPPS (*Lm*FPPS) but is replaced by tyrosine in both *T. cruzi* FPPS (*Tc*FPPS) and *T. brucei* FPPS (*Tb*FPPS) (Figure 11B). This conservative substitution of aromatic residues should not affect substrate chain elongation and the final product length.

### Bisphosphonate Inhibitors of Protozoan FPPSs and Their Target Binding

**Figure 12:**
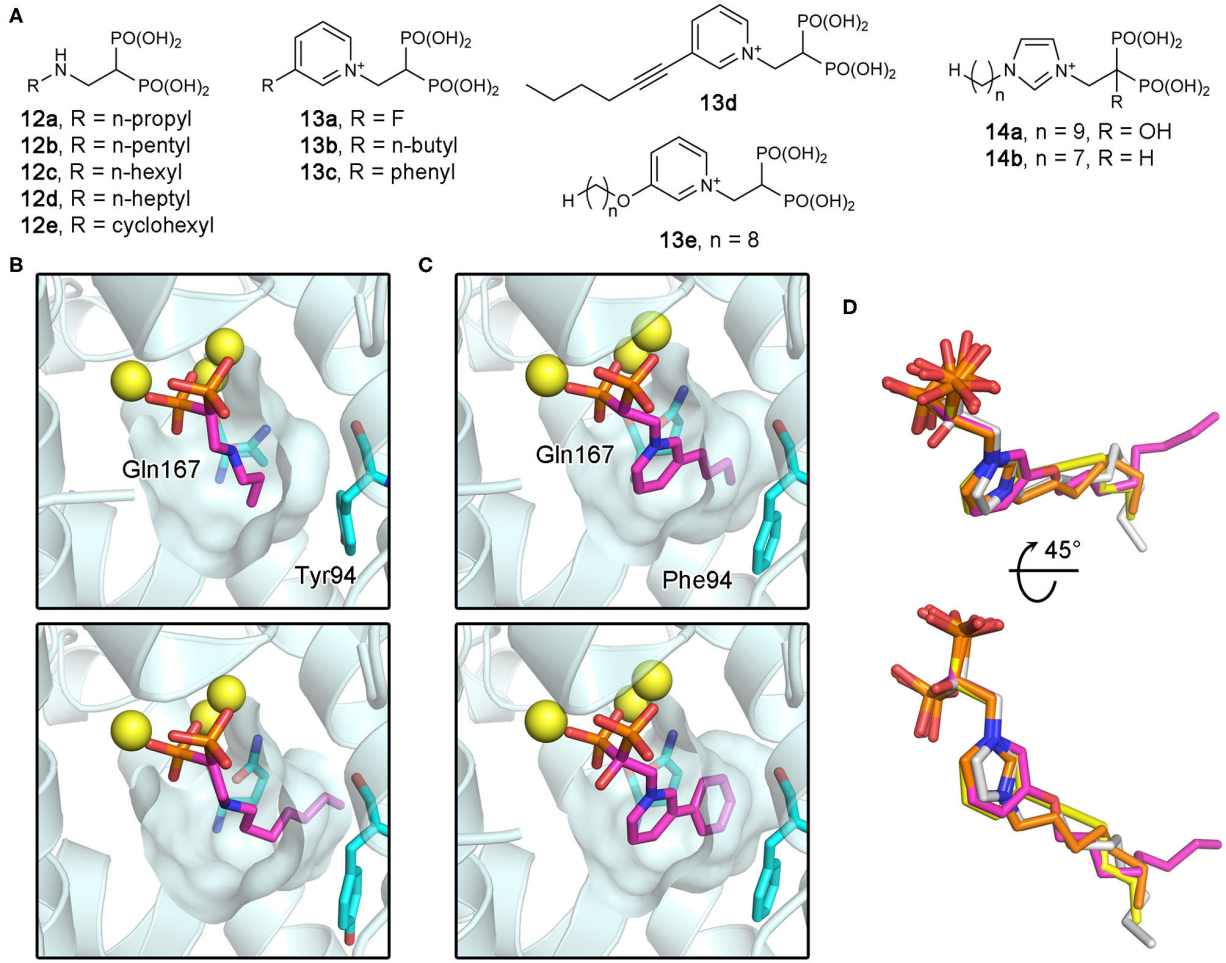
Bisphosphonate inhibitors of trypanosomatid FPPS and their target binding. **(A)** Examples of exploratory bisphosphonate inhibitors of trypanosomatid FPPS. **(B)** The binding of **12a** (top panel; PDB ID: 4DXJ) and **12d** (bottom; PDB ID: 4DWG) to *Tc*FPPS. The conformational changes in Tyr94 and Gln167 induced by **12d** results in an increase in the volume of the binding site hydrophobic pocket. **(C)** The binding of **13b** (top; PDB ID: 4JZX) and **13c** (bottom; PDB ID: 4JZB) to *Lm*FPPS. **(D)** Superimposed binding poses of **13d** (yellow; PDB ID: 5AEL), **13e** (magenta; PDB ID: 3EFQ), **14a** (white; PDB ID: 5AFX), and **14b** (orange; PDB ID: 5AHU).

As potential antiparasitic agents against *T. cruzi*, 2-alkylaminoethyl bisphosphonates have been explored. The representative compounds **12a–e** (Figure 12A) strongly inhibit the growth of the most clinically relevant form of the organism, with their IC_50_ values against *Tc*FPPS in the low nanomolar to micromolar range (Szajnman et al., 2003, 2008; Rosso et al., 2011). Crystallographic analysis (Aripirala et al., 2012) showed that the bisphosphonate backbones of these inhibitors make identical binding interactions with *Tc*FPPS (i.e., metal ion-mediated electrostatic interactions to the DDXXD motifs; as also observed for the human enzyme). Deeper in the active site, however, the binding modes of these compounds vary slightly. With **12c** and **12d**, the side chains of Tyr94 and Gln167 adopt different conformations to accommodate the longer N-alkyl groups of these inhibitors (bottom panel, Figure 12B). The n-heptyl group of **12d**, in particular, reaches the back end of the active site hydrophobic cavity. The additional van der Waals contacts created here contribute to the higher binding affinity (*K*_d_ = 58.8 nM) and thus inhibitory potency (IC_50_ = 58.0 nM) compared to those of **12c** (*K*_d_ and IC_50_ = 400 and 490 nM, respectively) (Szajnman et al., 2008; Aripirala et al., 2012). Binding characterization by isothermal titration calorimetry (ITC) also provided insightful findings (Aripirala et al., 2012). The binding of **12a**-**d** was shown to be entropically driven, where the unfavorable loss of conformational freedom is offset by the favorable gain of entropy from the burial of the hydrophobic alkyl chains. While ITC data for **12e** could not be obtained in this study, the high potency of this inhibitor (IC_50_ = 13 nM) can be explained based on the same thermodynamic principle. The conformational flexibility of the cyclohexyl ring is significantly more limited than the n-hexyl chain, and therefore, the binding of **12e** allows the burial of a comparably large hydrophobic surface without the loss of conformational entropy experienced by **12d**.

Subsequently, similar binding studies have been carried out with the pyridinium and imidazolium bisphosphonates **13a–e** and **14a**, **b** (Figure 12A). The binding of **13a–c** was characterized against *Lm*FPPS (Aripirala et al., 2014), which showed analogous interactions to those observed with *Tc*FPPS earlier. Notably, the side chains of Phe94 (Tyr94 in *Tc*FPPS) and Gln167 move to accommodate the bulkier n-butyl and phenyl groups of **13b** and **13c** (Figure 12C). As a single-digit nanomolar inhibitor (IC_50_ = 9 nM), **13c** showed the highest potency against *Lm*FPPS among the tested compounds (Sanders et al., 2005). This study also identified differences between *Lm*FPPS and hFPPS that could potentially be exploited to design inhibitors that are more specific toward the parasitic homolog. The residues Leu129 and Thr164, which are located at the backside of the active site hydrophobic cavity, are replaced by Asn133 and Glu168 in hFPPS (Ile129 and Ala164 in *Tc*FPPS). An additional hydrophobic group substituted at the distal ring of **13c**, for example, may allow more favorable binding to *Lm*FPPS. Studies of *Tb*FPPS-bisphosphonate binding have led to similar findings (Zhang et al., 2009; Yang et al., 2015). The most notable difference was in the positions of the inhibitors' side chain termini, with **13e** adopting a clearly different binding pose compared to those of the shorter-chain analogs (i.e., **13d**, **14a**, and **14b**) (Figure 12D). The structural data indicate that analogs with longer side chains would be poor inhibitors of *Tb*FPPS due to increased steric hindrance with the binding site residues. Inhibitor **13d** showed the highest potency, which is likely related to the fact that its 6-carbon substituent on the pyridinium ring provides increased hydrophobicity without the full conformational flexibility of comparably sized n-alkyl chains.

Although the above bisphosphonates have clear inhibitory activity against the trypanosomatid parasites both *in vitro* and *in vivo*, the correlation between their potency for inhibiting FPPS and for inhibiting parasitic growth is extremely poor (Yang et al., 2015). The presence of other targets for bisphosphonates has been suggested as a possible explanation (Yang et al., 2015). Bisphosphonate inhibition of GGPPS (Mukkamala et al., 2008), squalene synthase (Shang et al., 2014), and farnesyl protein transferases (Holstein et al., 1998) has been reported, which is unsurprising based on the roles of these enzymes in the isoprenoid pathways; they all use a prenyl pyrophosphate substrate. In this light, it is interesting that bisphosphonates can also inhibit *T. cruzi* hexokinase (Hudock et al., 2006). Although very distantly related (Park and Gupta, 2008), enzymes of another sugar kinase class, adenosine kinase and ribokinase, are also inhibited by bisphosphonates (Park et al., 2006, 2007). Additional future work is required to determine whether there are other protozoan enzymes targetable by bisphosphonate compounds.

### Fragment-Based Discovery of Non-bisphosphonate Inhibitors of *Tb*FPPS and *Tc*FPPS

More recently, Novartis has identified non-bisphosphonate inhibitors of *Tb*FPPS (Munzker et al., 2020) and *Tc*FPPS (crystal structures have been deposited to the PDB) through fragment-based screening. Although the full discussion of this work is beyond the scope of the current review, as none of the discovered inhibitors is a phosphonate- or bisphosphonate-based compound, some of their key findings are worth highlighting here. Importantly, this work confirmed the presence of an allosteric site in *Tb*FPPS at an analogous location, as identified in hFPPS. Allosteric inhibition has only been reported for the human homolog previously. In addition, inhibitors that act at the active site but do not belong to the bisphosphonate class of compounds have been identified for the first time for any FPPS homolog. Furthermore, additional fragment binding sites that could potentially be exploited to inhibit the enzyme's open-to-closed conformational change and thus its catalytic activity have been discovered. It remains to be seen whether the newly discovered chemotypes can be developed into clinical drugs to treat tropical parasitic diseases.

## Insect FPPS

### FPPS as an Insecticidal Target

**Figure 13:**
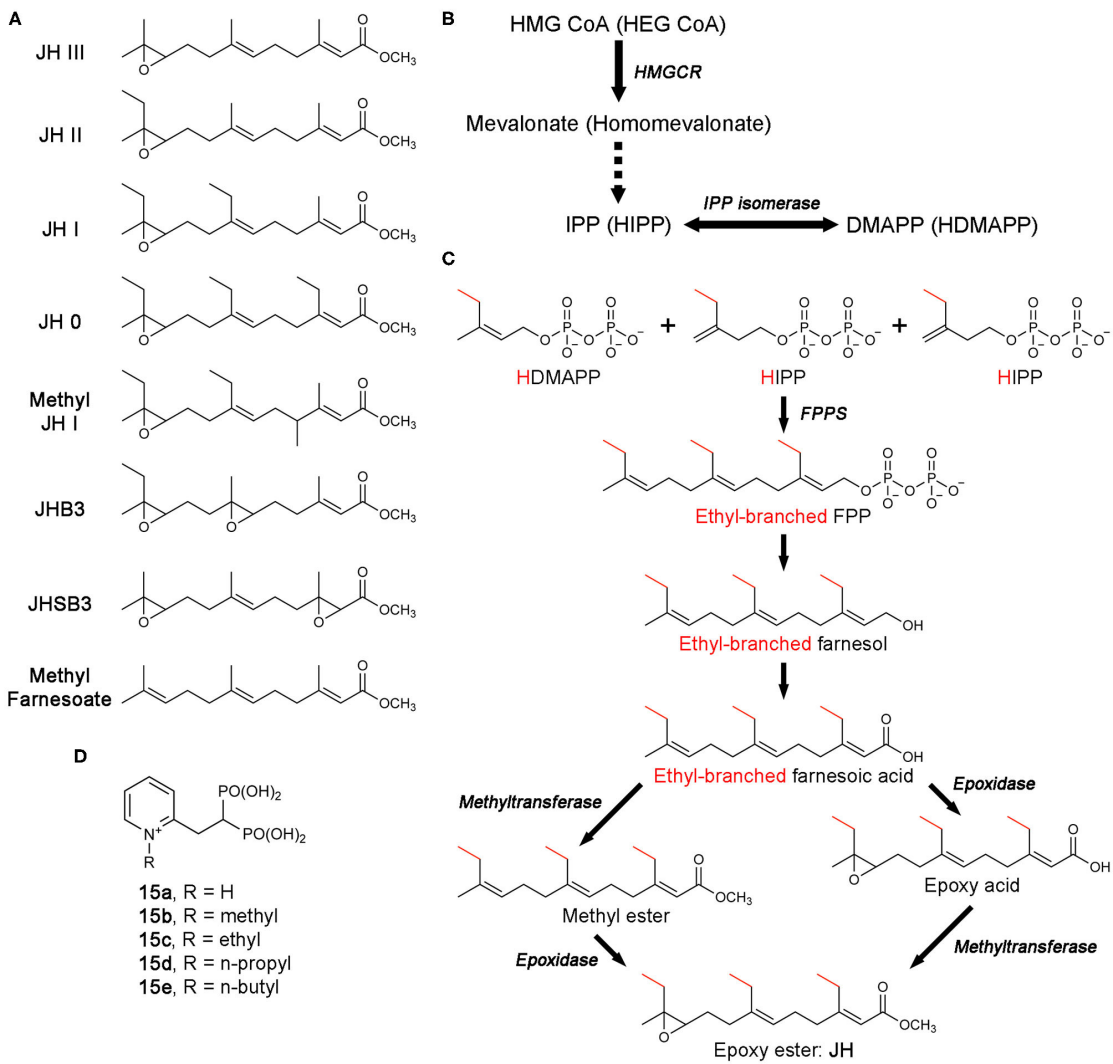
Biosynthesis of juvenile hormones and bisphosphonate inhibitors of *Cf*FPPS2. **(A)** Molecular structures of juvenile hormones (JHs). **(B)** The MVA pathway and its promiscuous ethyl-branched metabolites (in brackets). The intermediate steps catalyzed sequentially by mevalonate kinase, phosphomevalonate kinase, and mevalonate pyrophosphate decarboxylase are omitted for simplicity (dotted arrow). Abbreviations: HEG CoA, hydroxylethylglutaryl coenzyme A; HIPP, homoisopentenyl pyrophosphate; HDMAPP, homodimethylallyl pyrophosphate. **(C)** The production of FPP/ethyl-branched analogs of FPP and their conversion to JH. Various combinations of the starting substrates lead to different forms of JH. For example, the condensation of HDMAPP with HIPP and IPP produces 7,11-bishomofarnesyl pyrophosphate, which is then converted to JH I. Notably, methyl farnesoate is the immediate precursor of JH III and lacks the epoxide moiety characteristic of JH. While there is a long-standing debate, the potential role of methyl farnesoate as a JH has been recognized. **(D)** N-alkylated *ortho*-substituted bisphosphonate inhibitors of *Cf*FPPS2.

Found almost solely in insects, the gonadotropic and morphogenetic juvenile hormone (JH) is a compelling possible target for the development of insecticides (Williams, 1967). Characterized by its sesquiterpenoid (i.e., 15-carbon terpenoid) molecular backbone, JH serves many functions in the development and reproduction of insects, such as retention of larval structures, prevention of adult differentiation, and ovarian maturation (Cusson and Palli, 2000). Consequently, reduction in JH levels can result in improperly formed adults that may not be able to reproduce or survive. There are eight different forms of JH identified thus far, each with a distinct structure (Figure 13A) (Picard et al., 2018). All eight retain the sesquiterpenoid backbone, with JH III being the simplest and most ubiquitous. Also, JH III is the only JH found in the majority of insects. Species of the Lepidoptera, however, produce four other JHs that carry one or more additional carbons (JH 0, 4-methyl-JH I, JH I, and JH II; Figure 13A), which are unique to the order (Cusson and Palli, 2000).

The biosynthesis of JH starts from the MVA pathway (Figure 13B). The enzymatic steps up to the point where FPPS produces FPP (or its ethyl-branched homologs) are identical to those in humans (Figures 13B,C). However, the steps that follow are unique to JH synthesis, resulting in the conversion of FPP and its homologs to various forms of JH (Figure 13C) (Goodman and Cusson, 2012).

Unlike in most organisms, FPPS is not encoded by a single-copy gene in the Lepidoptera. Two distinct paralogs exist, type-I and type-II, also referred to as FPPS1 and FPPS2, respectively (Cusson et al., 2006). The tissue-specific role of FPPS1 and FPPS2 has yet to be fully determined. However, transcription analysis in *Bombyx mori* (i.e., domestic silk moth; silkworm as larvae) has shown that while FPPS1 is ubiquitously expressed, FPPS2 is expressed more specifically in the endocrine glands known as the corpora allata, the exclusive site of JH production and release (Cusson et al., 2006; Kinjoh et al., 2007). In addition, analysis of prenyltransferase activity in homogenates of corpora allata from *Manduka Sexta* (tobacco hawk moth; tobacco hornworm as larvae) was found to have a preference for HDMAPP, the ethyl-branched homolog of DMAPP, over DMAPP itself (Sen et al., 1996). These observations support the role of FPPS2 in JH biosynthesis and suggests that of the two paralogs, type-II may be more specialized for the production of ethyl-branched FPP homologs.

In an effort to identify compounds that can disrupt insect development by inhibiting JH biosynthesis, Sen and colleagues have created a number of new bisphosphonate-based FPPS inhibitors (Sen et al., 2015). Molecular docking employing a homology model of *Choristoneura fumiferana* FPPS2 (*Cf*FPPS2) has led to the selection of N-alkylated *ortho*-substituted pyridinium bisphosphonates as candidates for selective inhibition of this enzyme (**15a**–**e**; Figure 13D). Commonly referred to as the spruce budworm, *C. fumiferana* is one of the most destructive pests of conifers in the eastern North America (Nealis, 2016) and therefore represents an appropriate insecticidal target. *In vitro* enzyme assays have confirmed the specificity of the new inhibitors toward *Cf*FPPS2 over other insect and mammalian FPPS homologs (Sen et al., 2015). This result supported the notion that there could be considerable steric latitude in the active site of *Cf*FPPS2 exploitable for drug discovery.

### Overall Structure of *Cf*FPPS2

**Figure 14:**
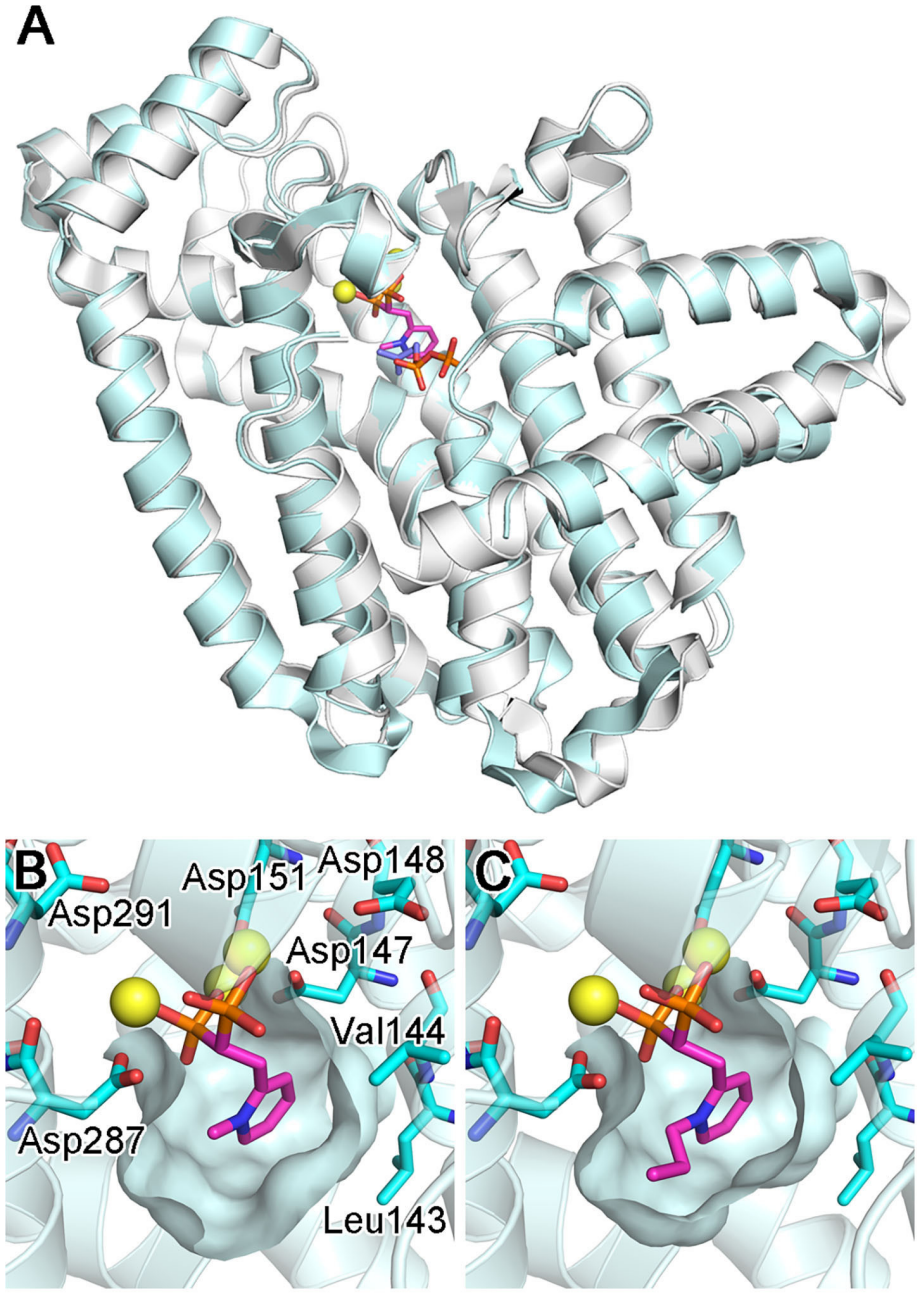
Structures of bisphosphonate-bound *Cf*FPPS2. **(A)** The overall structure of the *Cf*FPPS2-**15b**-IPP ternary complex (cyan; PDB ID: 6B06). Superposed in white is the structure of a human FPPS ternary complex (PDB ID: 4H5E). **(B)** The binding of inhibitor **15b**. The structure is of the *Cf*FPPS2-**15b** binary complex (PDB ID: 6B04). **(C)** The binding of inhibitor **15d** (PDB ID: 6B07). Displayed residues are identical as in **(B)**.

Crystal structures of *Cf*FPPS2 have been determined recently, one in its apo form and others in complex with the N-alkylated pyridinium bisphosphonates **15b** and **15d** (Picard et al., 2018). The overall structure of this enzyme is highly similar to those of the other structurally characterized FPPS homologs, including the human and protozoan parasite enzymes (Figure 14A). The core of *Cf*FPPS2 is also composed of a 10-helix bundle that contains a central catalytic cavity. The 3D arrangement of the allylic and homoallylic substrate binding sites, as well as the two aspartic acid-rich motifs (^147^DDIMD^151^ and ^287^DDYLD^291^), are fully conserved. The three-helix insert that forms a small peripheral domain is present as well.

### Inhibitor Binding and Implications for Future Drug Design

As expected from earlier structures of other bisphosphonate-bound FPPS homologs, inhibitors **15b** and **15d** bind to the allylic substrate site of *Cf*FPPS2 (Picard et al., 2018). The binding interactions to the bisphosphonate moiety are largely identical to those previously observed; two Mg^2+^ ions mediate the interactions to the first DDXXD motif on one side, and another Mg^2+^ ion to the second DDXXD motif on the other (Figure 14B). The bisphosphonate moiety also interacts with the conserved residues Arg156, Lys244, and Lys301 (analogous to Arg112, Lys200, and Lys257 of the human homolog) in forming direct salt bridges. The side chain pyridine of **15b** and **15d** partially fills the hydrophobic pocket that accommodates the growing isoprene chain during catalysis (Figures 14B,C). Surprisingly, however, the N-alkyl groups on their pyridine moiety extend not into this hydrophobic pocket but into the IPP binding site (Figures 14B,C). This binding mode contradicts the results of the previous docking study (Sen et al., 2015), which showed the alkyl groups of these inhibitors pointing toward Leu143 and Val144, away from the IPP binding site. Notably, Leu143 replaces one of the capping phenyls in the human enzyme (i.e., Phe99; the residue at this location is either phenylalanine or tyrosine in the trypanosomatid enzymes). This difference leads to a slightly increased volume of the allylic site hydrophobic pocket in *Cf*FPPS2 and may contribute to the enzyme's substrate promiscuity (i.e., for the ethyl-branched homologs of DMAPP and GPP). Between the two inhibitors **15b** and **15d**, the mode of binding is almost identical. **15d** binds to *Cf*FPPS2 in the same manner as **15b**, except that its larger n-propyl group extends further into the IPP binding site (Figure 14C).

Based on the structural information they obtained, Picard and colleagues were able to propose potential modifications to improve their inhibitors (Picard et al., 2018). Most notably, the N-alkyl groups of **15b** and **15d** bind into the IPP binding pocket, where the pocket is lined with several polar and charged residues. Therefore, increasing the hydrophobicity of the alkyl group is likely to destabilize this particular binding mode. This prediction is supported by the fact that the IC_50_ of **15d**, which contains the n-propyl group, is higher than that of **15b**, which instead contains the methyl group (1.7 vs. 0.5 μM) (Sen et al., 2015). It is postulated that replacement of the propyl group with a methyl or ethyl moiety containing a charged or H-bond-donating substituent may provide additional favorable binding interactions (Picard et al., 2018). Attachment of an additional, lipophilic group at the *para* position to the pyridine nitrogen, which would fill the chain-elongation hydrophobic pocket, may also give rise to improved inhibitors.

## Conclusion

Structure-based drug design approaches provide faster and more cost-efficient means of lead discovery than the traditional methods, such as high-throughput screening (Batool et al., 2019). The ultimate limitation of these approaches is that they require detail knowledge of the target molecule's 3D structure. With ongoing technological advancements in the methods of molecular biology and macromolecular structure determination, and together with the striking progress being made in genomics and proteomics, increasing numbers of drug targets are becoming available for structure-based approaches. Identification of binding pockets in therapeutic targets and analysis of the molecular interactions between the targets and lead inhibitors have now become a fundamental part of medicinal chemistry efforts, whether for academic researches or industrial projects. The story of FPPS is a testimonial to this drug discovery paradigm shift. The first bisphosphonate drugs were developed in the traditional way (which relies on phenotypic screening; forward pharmacology) based on the serendipitous discovery of antiresorptive effects of inorganic pyrophosphate (Russell, 2011). Since then, the molecular target of the bisphosphonate drugs remained unknown for decades. Now, ~200 structures of FPPS from different organisms are available, which show a variety of bound ligands, including the best current N-BP drugs and non-bisphosphonate allosteric inhibitors. These structures have allowed us a greater understanding of the enzyme's catalytic cycle and its product feedback mechanism. They have also provided crucial insights into inhibitor binding and subsequent drug discovery strategies. In this review, we have tried to summarize the great wealth of structural information currently available on FPPS. Application of this information will likely lead to the identification of additional novel inhibitors of FPPS that may prove useful as clinical agents.

## Author Contributions

All authors listed have made a substantial, direct and intellectual contribution to the work, and approved it for publication.

## Conflict of Interest

The authors declare that the research was conducted in the absence of any commercial or financial relationships that could be construed as a potential conflict of interest.
